# Immune synapse in the tumor microenvironment: functional dynamics and clinical translational perspectives

**DOI:** 10.3389/fimmu.2026.1983341

**Published:** 2026-10-27

**Authors:** Yujie Ge, Senxu Lu, Bingqian He, Vivek P. Chavda, Jianli Gao

**Affiliations:** 1 College of Pharmacy, Zhejiang Chinese Medical University, Hangzhou, China; 2 Academy of Chinese Medical Sciences, Zhejiang Chinese Medical University, Hangzhou, China; 3 Department of Pharmaceutics and Pharmaceutical Technology, Lallubhai Motilal (L. M.) College of Pharmacy, Ahmedabad, Gujarat, India; 4 Zhejiang Key Laboratory of Blood-Stasis-Toxin Syndrome, Zhejiang Chinese Medical University, Hangzhou, China; 5 Key Laboratory for Diagnosis and Treatment of Upper Limb Edema and Stasis of Breast Cancer, Hangzhou, Zhejiang, China

**Keywords:** immune synapse, tumor, clinical treatment, cytoskeleton, synaptic function

## Abstract

Immune synapse (IS) is a dynamic supra-molecular structure between immune cells and antigen-presenting cells (APCs) or other target cells. Through precise spatiotemporal organization, the IS integrates antigen recognition, signal amplification, and effector function, and is thereby associated with, and may contribute to the initiation, intensity, and durability of antitumor immune responses. Within the tumor microenvironment (TME), however, the structural integrity and signaling function of the IS are frequently disrupted by molecular, metabolic, physical, and epigenetic abnormalities, which may represent an important barrier to effective immunotherapy. This review dissects the regulation of IS in tumors from a multiscale perspective. At the molecular level, we discuss the LFA-1/ICAM-1 adhesion axis, co-stimulatory/co-inhibitory signals, neuroimmune crosstalk, and supramolecular assembly driven by liquid–liquid phase separation. At the physical level, we address the roles of matrix stiffness, tumor cell membrane mechanics, and mechanotransduction in IS assembly and effector function. At the metabolic and post-translational levels, we summarize how glycosylation remodeling, cholesterol metabolism, lactate accumulation, and adenosine signaling induce the synaptic signaling activation and cytoskeletal reorganization. We further summarize IS-targeted therapeutic strategies, including adhesion molecule agonists, immune checkpoint blockade, synaptic remodeling of engineered immune cells, metabolic modulation, and nanoengineering, and evaluate their translational potential and limitations. Finally, we propose that a paradigm shift from “repairing the synapse” to “programming the synapse”—by integrating biochemical signals, biophysical properties, and neuroimmune regulation to achieve precise and reversible control of IS function—may overcome current barriers in solid tumor immunotherapy and provide a conceptual framework for IS-guided precision immunotherapy.

## 1 Introduction

The immune synapse (IS) is a specialized, dynamic, and highly ordered cell‐cell contact interface formed between immune effector cells, such as T cells and natural killer (NK) cells, and their cognate target cells (e.g., malignant tumor cells and virus‐infected cells) or antigen‐presenting cells (APCs). These supramolecular activation clusters (SMACs) assemble when immune effector cells recognize specific peptide‐major histocompatibility complex (pMHC) molecules presented on antigen‐presenting cells (APCs) or cognate target cells at the IS (1), serving as a platform for signal integration and directional secretion of effector molecules. Although the IS shares functional similarities with neuronal synapses in terms of spatially directed secretion and signal polarization, its primary role is the precise control of immune activation and the directional execution of effector functions. From a functional perspective, the IS operates as a dynamic nanomachine that integrates antigen recognition, co-signal transduction, cytotoxic polarization, and mechanical sensing. The integrity of this structure is increasingly recognized as a critical contributor to the initiation, intensity, and durability of antitumor immune responses (2).

The classical IS adopts a highly organized, concentric “bull’s-eye” architecture at the ultrastructural level. The central SMAC (cSMAC) is enriched with T cell receptor (TCR)–pMHC complexes and co-stimulatory molecules. The peripheral SMAC (pSMAC) contains abundant the integrin lymphocyte function-associated antigen 1 (LFA-1; αLβ2)and its ligand, intercellular adhesion molecule 1 (ICAM-1), which mediate firm adhesion (3). The distal supramolecular activation cluster (dSMAC) is characterized by the accumulation of filamentous actin (F-actin) and the phosphatase cluster of differentiation 45 (CD45), contributing to the dynamic turnover of microclusters. TCR microclusters are continuously generated, converge, and disassemble at the contact interface, serving as the basic units of IS structure and signal output. This process is driven primarily by retrograde actin flow, with signal strength progressively amplified and subsequently attenuated as microclusters transit toward the cSMAC. Such a precise molecular architecture ensures efficient signal transduction while also providing spatial coordinates for the directed secretion of effector molecules.

During tumor immune surveillance and the effector phase, the IS functions as a key convergence point that influences whether immune cells proceed to effective recognition and elimination of malignant cells. Through TCR–pMHC interactions, the IS achieves antigen recognition at the single-cell level with both high sensitivity and stringent specificity, enabling the reliable discrimination of neoantigens arising from mutated genes from normal self-antigens (4). Simultaneously, the IS serves as the central platform for the spatiotemporal competition between co-stimulatory signals (e.g., CD28–CD80/86) and co-inhibitory signals (e.g., programmed cell death protein 1 (PD-1)–programmed death-ligand 1 (PD-L1)). The stability and molecular composition of the IS are associated with and may influence whether immune cells proceed toward full activation, tolerance, or exhaustion (5). During the effector phase, the IS forms a specialized synaptic cleft that directs cytotoxic granules containing perforin and granzyme B to polarize along the microtubule-organizing center (MTOC) toward the contact interface and undergo precise exocytotic release (3). This polarized secretory mechanism ensures that cytotoxic effects remain spatially confined, thereby minimizing collateral damage to adjacent healthy tissues. Serial killing capacity further depends on the dynamic disassembly and reassembly of the IS. In addition, membrane lipid events—such as diacylglycerol (DAG)-induced negative membrane curvature and ectosome shedding—enable cytotoxic T lymphocytes (CTLs) to rapidly detach from target cells after granule release and reorient toward the next target (6).

Recent studies have demonstrated that the IS is not merely a biochemical signaling platform but also a sophisticated mechanical sensor. T cells exert piconewton-scale forces through TCR–pMHC bonds and sense physical parameters such as antigen affinity, target cell stiffness, and membrane curvature. They then convert these mechanical stimuli into biochemical signals via mechanotransduction—the process by which mechanical forces are translated into intracellular signaling events (7). This capacity, termed mechanosensing, allows T cells to probe the physical properties of their targets. This mechanical sensing capacity is essential for T cells to overcome the physical barriers imposed by the high interstitial pressure of solid tumors and to sustain effective recognition and killing. Solid tumors frequently attenuate T-cell mechanical responses by altering matrix stiffness and tumor cell membrane fluidity, or by disrupting TCR–pMHC force transmission, thereby establishing a “mechanical immune checkpoint”. Accordingly, restoring the mechanical sensing function of the IS is emerging as a promising direction for solid tumor immunotherapy (8).

In summary, the assembly quality, signal composition, dynamic behavior, and mechanical sensing capacity of the IS are key features associated with and likely contributing to antitumor immune responses. A deeper understanding of the molecular architecture and regulatory networks of the IS is key to unraveling the mechanisms of tumor immune evasion and to establishing the theoretical basis for next-generation IS-targeted immunotherapies. Unlike previous reviews that focus on individual IS components, this review makes four contributions. First, we integrate molecular, metabolic, biophysical, and neuro-immune regulation into a single multi-scale framework. Second, we highlight residual positive mechanisms that sustain IS formation in the immunosuppressive TME, which prior reviews often overlook. Third, we evaluate IS-targeted therapies through an efficacy–toxicity–delivery trilemma, rather than efficacy alone. Fourth, we propose a shift from “repairing the synapse” to “programming the synapse”, incorporating biochemical, biophysical, and neuro-immune parameters. We also outline how advanced imaging and AI-driven IS phenotyping can inform patient stratification and discuss the analytical and clinical validation steps required before clinical deployment.

## 2 Literature search strategy

This narrative review was based primarily on a literature search conducted in PubMed/MEDLINE, Web of Science, and ClinicalTrials.gov from database inception to October 1, 2026. Reference lists of key articles and reviews were also manually screened. Clinical trial information was verified through ClinicalTrials.gov where applicable. Search terms were combined into thematic blocks. Core IS terms included “immune synapse”, “immunological synapse”, “T cell receptor”, “TCR signaling”, “supramolecular activation cluster”, “cSMAC”, “pSMAC”, “dSMAC”, “TCR microcluster”, “immune synapse architecture”, and “kinapse”. Tumor microenvironment and immunotherapy terms included “tumor microenvironment”, “cancer immunotherapy”, “checkpoint blockade”, “PD-1”, “PD-L1”, “CTLA-4”, “CAR-T”, “CAR-NK”, “TCR-T”, “bispecific T-cell engager”, “T cell engager”, and “adoptive cell therapy”. Neuro-immune terms included “neuro-immune crosstalk”, “neuro-immune synapse”, “cancer neuroscience”, “neurotransmitter”, “sympathetic”, “parasympathetic”, and “substance P”. Imaging and AI terms included “super-resolution microscopy”, “STED”, “STORM”, “lattice light-sheet microscopy”, “TIRFM”, “FRET”, “LIPSTIC”, “machine learning”, “deep learning”, “artificial intelligence”, “CAR-T IS quality”, and “imaging flow cytometry”.

Selected publications included original research articles, clinical trials, and authoritative reviews addressing IS structure, signaling, assembly, or function in tumor immunity; studies on CAR-T, TCR-T, CAR-NK, bispecific antibodies, and T cell engagers relevant to IS formation or disruption; studies on TME-mediated IS dysregulation, including metabolic, biophysical, and neuro-immune mechanisms; studies on advanced imaging technologies and AI-assisted IS analysis; and clinical trial reports and regulatory documents for IS-targeted therapies. Given the broad scope of the review, no restrictions were applied based on study design or host species. Studies unrelated to the scope of the review were excluded, but articles not specific to the IS were included selectively when they provided relevant biological context for interpreting findings associated with IS biology and were clearly identified in the text. As this is a narrative rather than a systematic review, no formal risk-of-bias assessment or synthesis of quantitative evidence was performed.

## 3 Mechanisms of immune synapse formation and cell type specificity in tumors

The mature IS typically adopts a characteristic concentric “bull’s-eye” architecture in two-dimensional projections, comprising three functional zones from the center outward: the central supramolecular activation cluster (cSMAC), the peripheral supramolecular activation cluster (pSMAC), and the distal supramolecular activation cluster (dSMAC) (**Figure 1**). This canonical model was first described by Monks and colleagues in T cell–B cell conjugates and has since been corroborated by numerous super-resolution and electron microscopy studies.

**Figure 1:**
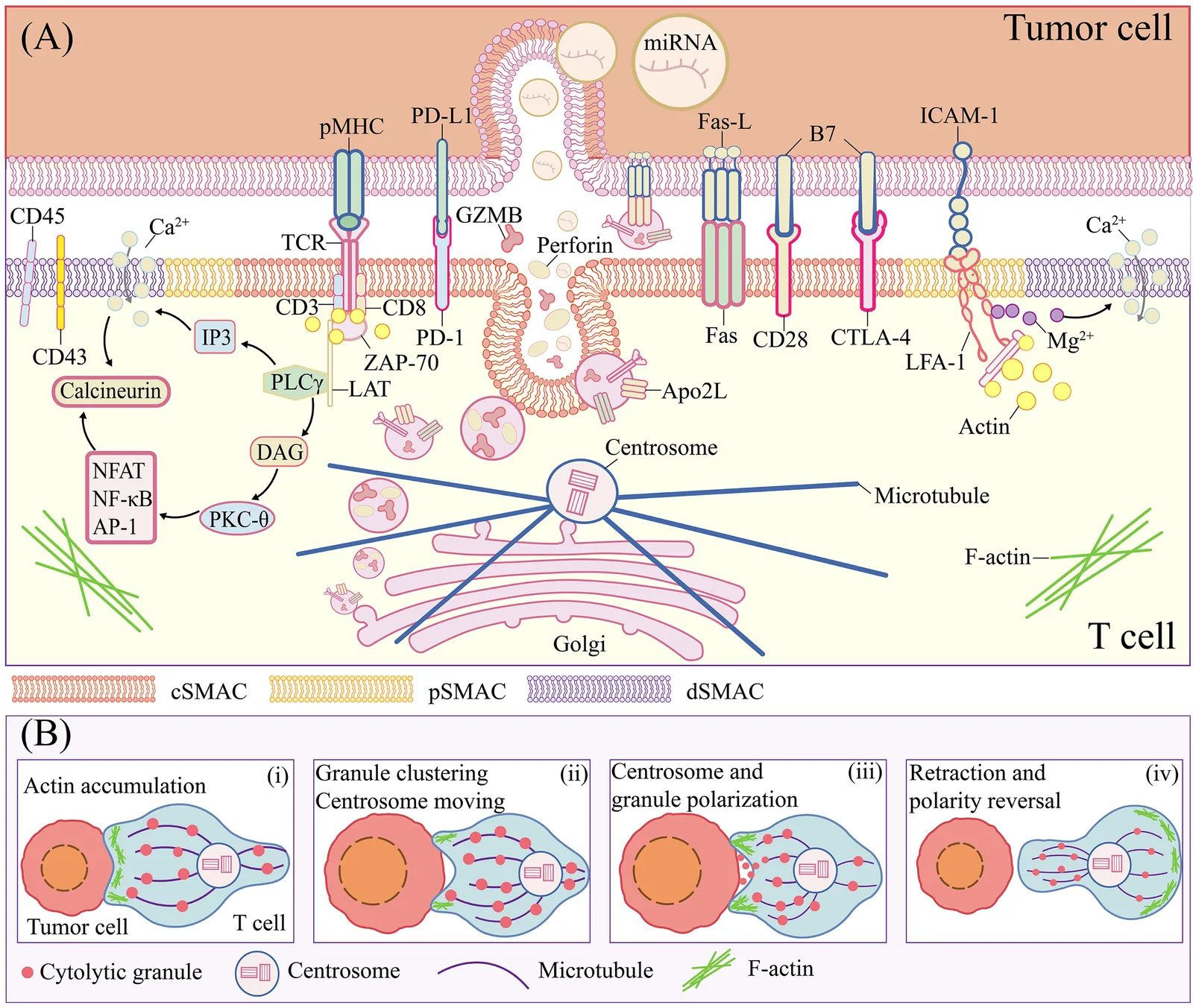
Schematic illustration of immune synapse formation and cytolytic granule polarization at the T cell–tumor cell interface. **(A)** Molecular architecture and signal transduction within the immune synapse. The T cell–tumor cell interface is organized into supramolecular activation clusters (SMACs), comprising central (cSMAC), peripheral (pSMAC), and distal (dSMAC) regions. Engagement of the T cell receptor (TCR) with peptide–MHC (pMHC), together with co-stimulatory, inhibitory, and death receptor–ligand pairs (e.g., CD28/B7, PD-1/PD-L1, Fas/FasL) and adhesion molecules (LFA-1/ICAM-1), triggers downstream signaling cascades, including the PLCγ–IP3–Ca^2+^–calcineurin–NFAT and DAG–PKC-θ pathways. Cytoskeletal remodeling, driven by microtubules and F-actin, facilitates polarization of the centrosome and Golgi apparatus, ultimately leading to exocytosis of perforin- and granzyme B (GZMB)-containing cytolytic granules into the synaptic cleft. *(The bottom legend defines the color-coding for the cSMAC, pSMAC, and dSMAC regions).* **(B)** Sequential events underlying cytolytic granule polarization and exocytosis. This dynamic process proceeds through four stages: (i) actin accumulation at the contact site; (ii) granule clustering and centrosome translocation toward the immune synapse; (iii) full polarization of the centrosome and cytolytic granules; and (iv) centrosome retraction and polarity reversal. cSMAC (red), pSMAC (yellow), and dSMAC (purple). Cytolytic granules, centrosome, microtubules, and F-actin are indicated by the symbols shown.

### 3.1 Classical architecture of the IS

The cSMAC is enriched with TCR/CD3 complexes (9), co-stimulatory molecules (e.g., CD28, CD2), protein kinase Cθ (PKC-θ), and Src family kinases such as Lck and Fyn (**Figure 1**). It has traditionally been regarded as the core region for antigen recognition and initial signal triggering (10, 11). However, time-resolved imaging studies have revealed that newly formed TCR signal microclusters are generated at the periphery of the pSMAC and initiate signaling there, with signal intensity progressively declining as microclusters move into the cSMAC. These observations suggest that the cSMAC also participates in signal termination and receptor recycling (3). This dynamic perspective has revised the earlier notion that the cSMAC simply serves as the “activation center”.

The pSMAC is enriched with the integrin LFA-1, its ligand ICAM-1, and cytoskeletal adaptor proteins, including Talin and kindlin-3 (**Figure 1**). By forming a dense adhesion ring, the pSMAC provides mechanical stability and establishes a boundary for signal compartmentalization (12). In CD8^+^ T cells, the transition of LFA-1 from a low-affinity to a high-affinity conformation is highly dependent on Mg^2+^ and on inside-out signals driven by the cytoskeleton. This conformational switch enhances Ca^2+^ influx and downstream signaling while also contributing to metabolic reprogramming, thereby augmenting the tumoricidal capacity of T cells (13). Preclinical evidence indicates that the functional adequacy of LFA-1 is closely associated with the antitumor efficacy of bispecific antibodies and CAR-T cells (14). Thus, the pSMAC adhesion ring is not merely a physical scaffold but also a potential intervention node for modulating T cell function and therapeutic responsiveness.

The dSMAC is characterized by the accumulation of large transmembrane phosphatases such as CD45 and bulky glycoproteins including CD43 and CD44. Its primary function is to exclude CD45 from the tight contact zone through steric hindrance, thereby preventing premature dephosphorylation of proximal TCR signaling molecules and preserving signal fidelity (15). Recent studies further indicate that CD45 exclusion depends on cytoskeleton-driven lateral sorting of membrane proteins, representing a key aspect of molecular reorganization during IS formation (16).

The assembly of the IS is not a single-step event but rather a highly dynamic, multi-stage process that can be broadly divided into four phases (17).

Initial contact and low-affinity adhesion. Prior to specific antigen recognition, LFA-1 on the lymphocyte surface engages ICAM-1 on the target cell or APC through low-affinity, reversible interactions, mediating rolling contact and antigen scanning. Upon TCR recognition of pMHC, inside-out signals rapidly activate LFA-1, inducing its extension into a high-affinity conformation. This triggers local F-actin remodeling and the formation of a peripheral actin ring (3). The actin ring demarcates the synaptic boundary while also facilitating polarization of the MTOC toward the contact interface, pre-positioning the spatial coordinates for subsequent secretory events.

Signal activation and TCR microcluster formation. Following TCR engagement with pMHC, Src family kinases Lck and Fyn are recruited and activated, phosphorylating immunoreceptor tyrosine-based activation motifs (ITAMs) on CD3 chains and subsequently recruiting zeta-chain-associated protein kinase 70 (ZAP-70). ZAP-70 in turn phosphorylates adaptor proteins such as linker for activation of T cells (LAT) and SH2 domain-containing leukocyte protein of 76 kDa (SLP76), leading to the formation of detectable TCR signal microclusters at the periphery of the contact zone. These microclusters represent the basic functional units of signal transduction within the IS; their density and lifetime directly determine downstream signal strength.

Polarized transport and centripetal flow. Myosin IIA couples with retrograde F-actin flow to drive the directed transport of TCR microclusters along the actin cytoskeleton toward the center of the contact area (18). Concurrently, the PI3K–mTOR pathway supports sustained T cell killing by regulating mitochondrial translocation toward the synaptic region and maintaining local energy supply (19). The microtubule network also undergoes marked polarization during this stage: the MTOC moves directionally toward the IS, guiding lytic granules containing perforin and granzyme B, as well as multivesicular bodies, to the secretory domain (20, 21). Under microscopy, cytotoxic granules originally dispersed throughout the cytoplasm can be clearly observed accumulating on the side proximal to the target cell—a process termed granule polarization. This polarization ensures that high concentrations of perforin and granzyme B are released exclusively within the narrow synaptic cleft. Perforin forms pores in the target cell membrane, allowing granzyme B to enter and initiate the apoptotic program, thereby achieving efficient and specific target cell killing (22, 23).

Steady-state structure formation and functional execution. As signal microclusters continue to converge, TCR microclusters coalesce in the central region to form the mature cSMAC, while the pSMAC adhesion ring is further stabilized by talin and other cytoskeletal proteins. This steady-state structure serves as a platform for signal integration while also constituting the ultimate site for the directed exocytosis of cytotoxic effector molecules. Notably, the mechanical environment plays an important regulatory role during this process. Increased target cell stiffness can enhance TCR mechanosensing, promoting TCR phosphorylation, F-actin reorganization, and ZAP-70/Src kinase activation, thereby increasing T cell cytotoxic activity *in vitro (*24). However, pathological extracellular matrix stiffening in solid tumors often impairs T cell infiltration and synaptic engagement, contributing to a “mechanical immune checkpoint”. Thus, the net effect of mechanical cues on antitumor immunity is context-dependent and remains to be fully elucidated (9, 25).

Upon completion of effector function, the IS enters a dissociation phase characterized by the disappearance of microtubule-associated protein tau (MAPT) from the synaptic region, restoration of actin density, and withdrawal of microtubules from the contact zone, indicating synaptic disassembly and recovery of cell motility (26, 27). This dynamic dissociation capacity is essential for CTLs to execute serial killing and migrate between different target cells.

Not all ISs exhibit the canonical bull’s-eye configuration. Based on the spatial distribution of accumulated molecules, ISs can be classified into three major morphologies: multifocal IS, bull’s-eye IS, and unstable contact (kinapse) (9) (**Figure 2**). Multifocal ISs are commonly observed during early activation or under low antigen density, when TCR microclusters fail to fully converge into a single cSMAC and instead persist as multiple dispersed signaling clusters. Bull’s-eye ISs represent fully mature synaptic structures. Kinapses are unstable, continuously sliding contact modes in which cells maintain a dynamic equilibrium between migration and signal maintenance (3). In the tumor microenvironment, an increased proportion of kinapses may reflect instability in T cell–target cell interactions, thereby reducing killing efficiency (28).

**Figure 2:**
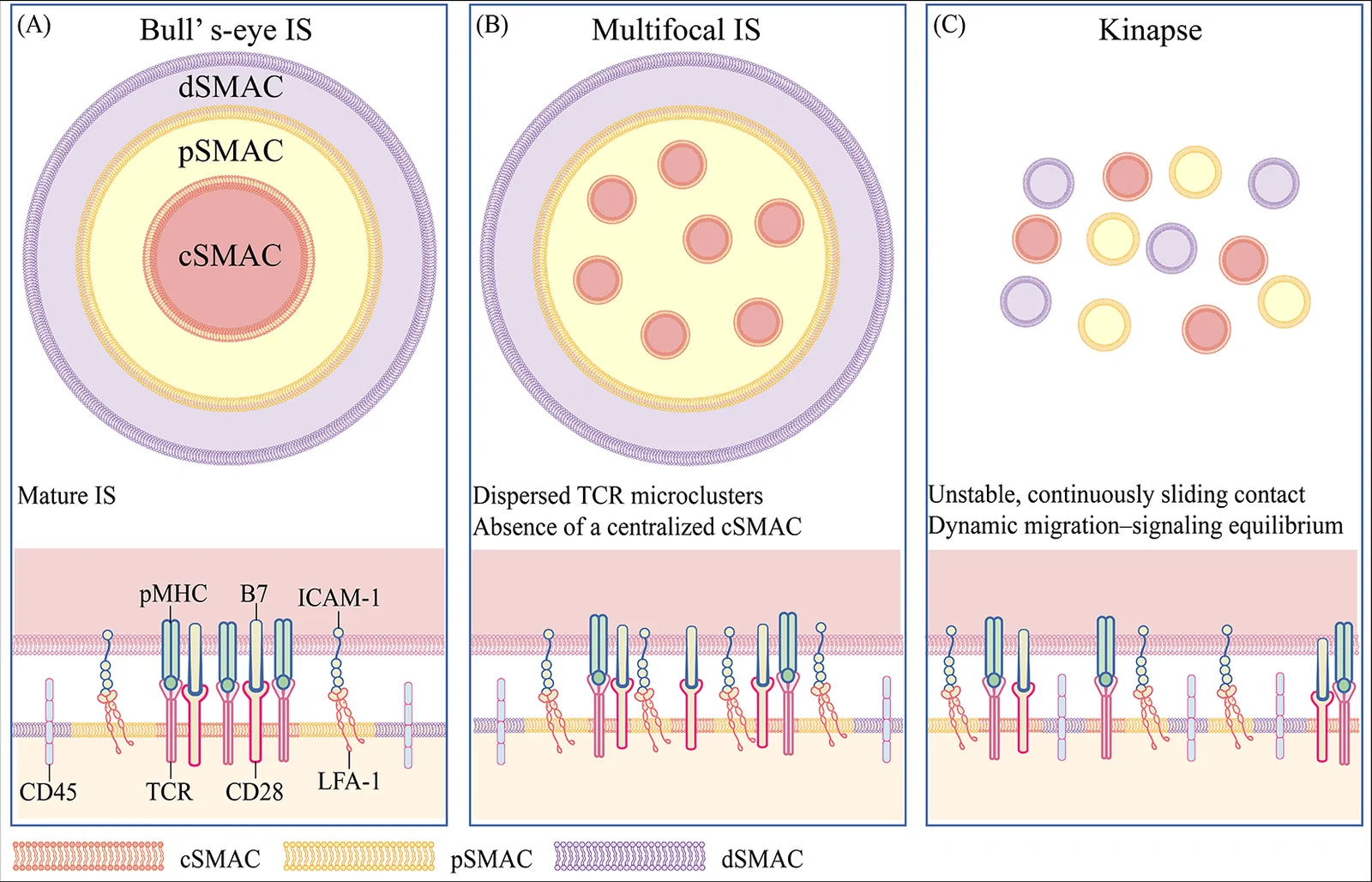
Schematic illustration of distinct spatial configurations of T cell immune synapses. The upper panels depict the planar topographic distribution of supramolecular activation clusters (SMACs), while the lower panels display the corresponding cross-sectional molecular profiles at the T cell–target cell interface. **(A)** Bull’s-eye IS (Mature IS): Exhibits a classic concentric compartmentalization into central (cSMAC), peripheral (pSMAC), and distal (dSMAC) regions. This architecture facilitates high-affinity, long-lasting receptor clustering (e.g., TCR, CD28) and robust sustained signaling. **(B)** Multifocal IS: Characterized by the absence of a centralized cSMAC and the presence of dispersed TCR microclusters, which support a diffuse signaling modality. **(C)** Kinapse: Represents an unstable, continuously sliding contact interface with scattered receptor-ligand distributions. This configuration allows motile T cells to maintain a dynamic balance between interstitial migration and transient antigen-triggered signaling (migration–signaling equilibrium). (The bottom legend defines the color-coding for the cSMAC, pSMAC, and dSMAC regions).

According to functional differences, ISs can also be divided into cytotoxic ISs and signaling ISs. The former are predominantly observed in the contacts of CTLs and NK cells with target cells and are characterized by directed lytic granule secretion and rapid killing. The latter are commonly found between helper T cells and APCs, with an emphasis on sustained cytokine secretion and signal integration (3). The functional orientation of the IS may differ across tumor types and treatment conditions, providing a potential basis for IS-based therapeutic response assessment and patient stratification.

In summary, the classical bull’s-eye architecture and dynamic assembly process of the IS constitute the molecular foundation of antitumor immune responses. Tumor cells can impair IS assembly or disrupt its functional balance by modulating the LFA-1/ICAM-1 adhesion axis, interfering with TCR microcluster formation, or altering the local mechanical environment. A comprehensive understanding of IS architecture and assembly dynamics is therefore essential for elucidating the cell biological mechanisms of tumor immune evasion and for designing IS-targeted therapeutic strategies, as discussed in the following sections.

### 3.2 Cell type-specific features of the immune synapse: structural and functional heterogeneity

The IS is not exclusive to T cells; rather, it represents a common intercellular communication strategy employed by diverse immune cells during target recognition or antigen presentation. Different immune cell types construct ISs with distinct structural and functional features, thereby playing differential roles in tumor immune surveillance and effector responses. Understanding these cell type-specific synaptic mechanisms is of considerable clinical significance for the precise targeting of the IS to enhance antitumor immunity.

#### 3.2.1 CD8^+^ T cell cytotoxic IS: granule polarization and supramolecular attack particles

CD8^+^ T cells typically form a classical “bull’s-eye” IS upon recognition of peptide–MHC (pMHC) on the tumor cell surface, characterized by mature cSMAC/pSMAC/dSMAC organization (**Table 1**). However, cSMAC formation is not a prerequisite for cytotoxic function. Effector CD8^+^ T cells can efficiently polarize cytotoxic granules and kill tumor cells even in the absence of a canonical cSMAC. This indicates that the synaptic architecture required for the delivery of cytotoxic payloads may be structurally less elaborate than the classic bull’s-eye, and that granule polarization itself constitutes the critical rate-limiting step for killing (39, 40).

**Table 1:** Comparative features of immune synapse architectures across different immune cell lineages.

Cell type	IS type	Contact cell type	Effector mechanism	Distinctive features
CD8^+^ T cells	Bull’s-eye cytotoxic IS	Tumor cells/infected cells	Perforin–granzyme axis; Fas–FasL	TCR-dependent signaling; CTLs form transient, mature IS (29).
CD4 T cells	Bull’s-eye secretory IS	APCs/other immune cells	Cytokine secretion; immunoregulation	Long-lived IS requiring continuous TCR signaling (30); primarily regulatory role (29).
Treg cells	Bull’s-eye signal transduction/antigen-presenting IS	APCs/other immune cells	Cytokine secretion; immunosuppression	Form stable, rapidly maturing immunosuppressive ISs (31).
B lymphocytes	Bull’s-eye antigen-extraction and antigen-presenting IS	Infected cells/tumor cells	Antigen capture for presentation to T cells	IS enriches BCR and excludes inhibitory receptors (e.g., CD45) (32).
CAR-T cells	Nonclassical multifocal cytotoxic IS	Tumor cells	Perforin–granzyme axis	Fast signal transmission but short duration; strong antigen binding but weak signaling (33).
NK cells	Bull’s-eye lytic IS	Tumor cells	Perforin–granzyme axis; Fas–FasL	Can form inhibitory synapses.
CAR-NK cells	Multifocal lytic IS	Tumor cells	Perforin–granzyme axis; Fas–FasL	Disordered synapse with punctate CAR:antigen clusters (34); irregular multifocal Lck arrangement (35).
Phagocytic cells	Phagocytic immune synapse	T cells	Present MHC class II–peptide complexes to TCRs	Promote T cell activation and enhance the interaction between innate immune and adaptive immune cells.
DCs	Signal transduction IS	T cells	Antigen presentation and T cell activation	TCR–MHC–peptide clusters in the center, CD28–B7 clusters in the periphery; transfer tumor antigens between DCs at synaptic contacts (36); enhance CD8^+^ T cell activation via cross-presentation following productive IS formation with CD4^+^ T cells (37).
cDC1	Signal transduction IS	CD8^+^ T	Cross-presentation to CD8^+^ T cells	CD8α^+^ cDC1 subset superior at stimulating CD8^+^ T cell immunity; cDC2 transfers tumor antigens to cDC1 via vesicles; resident CD8α^+^ DCs accumulate antigen and prime CD8^+^ T cells (36).
cDC2	Signal transduction IS	CD4^+^ T	Antigen presentation to CD4^+^ T cells	CD11b^+^ cDC2 subset better at stimulating CD4^+^ T cells; only migratory CD11b^+^ DCs effective at inducing CD4^+^ T cell proliferation (36).
Vγ9Vδ2 T cells	Bull’s-eye cytotoxic IS	Tumors cells	Perforin–granzyme axis; Fas–Fas	Dual-receptor–triple-ligand model: γ9δ2 TCR and CD277 interact with three ligands (BTN2A1, BTN3A1, and an unknown ligand) (38).

Recent studies have revealed that CD8^+^ T cells release both soluble perforin and pre-assembled supramolecular attack particles (SMAPs) composed of a perforin/granzyme core into the synaptic cleft (41). These particles are coated with thrombospondin-1 (TSP-1) or TSP-4 and act directionally on target cells within the synaptic cleft, markedly improving killing efficiency and spatial control (41). Furthermore, activated CD8^+^ T cells can locally express CD40L at the synapse, which engages CD40 on the target cell surface and can trigger apoptosis in selected tumor models, at least in part through caspase-8 activation (42). More broadly, perforin released at the synapse facilitates granzyme entry and may also trigger caspase-1 activation within the target cell, leading to gasdermin E (GSDME)-dependent pyroptosis, thereby extending synaptic killing from apoptosis to inflammatory cell death modalities (43). These findings suggest that CD8^+^ T cell synaptic cytotoxicity relies not solely on the classical perforin/granzyme pathway but also integrates multiple cooperative killing modes, offering new targets for strategies to enhance CTL killing efficacy.

#### 3.2.2 CD4^+^ T cell and Treg IS: stability, signal streamlining, and immunosuppression

CD4^+^ T cells form an IS structurally similar to that of CD8^+^ T cells, but with greater stability that can be maintained for several hours, a feature crucial for T cell fate determination (10) (**Table 1**). Stable IS formation promotes the differentiation of CD4^+^ T cells into functional subsets such as Th1, Th2, and Th17, and supports the generation of memory T cells (40). Interestingly, CD4^+^ T cells can acquire peptide–MHC class II (pMHC II) complexes from the plasma membrane of antigen-presenting cells (APCs), thereby adopting an antigen-presenting phenotype and becoming “T presenting cells” (Tpres) (20). Tpres cells predominantly differentiate into regulatory T cells (Tregs), whereas T cells stimulated by Tpres cells preferentially differentiate into Th17 cells (44). In the tumor microenvironment, this mechanism may be hijacked by tumor cells to expand Tregs through the promotion of Tpres generation, thereby suppressing antitumor immunity.

Tregs assemble a more stable bull’s-eye IS than conventional CD4^+^ T cells, with stability conferred by distinct signal transduction cascades and specialized cytoskeletal dynamics (31). Following TCR engagement, Tregs exhibit attenuated Ca^2+^ influx and ERK phosphorylation, yet signaling molecules including Lck, LAT, phospholipase C gamma 1 (PLCγ1), ZAP-70, and PKC-θ remain indispensable for their suppressive function (45). Compared with effector T cells, the IS of Tregs shows shorter recruitment duration and lower abundance of CD45, Lck, SLP76, and phosphorylated extracellular signal-regulated kinase (pERK); moreover, the co-stimulatory molecule B7 is recruited exclusively at the Treg IS, suggesting a streamlined signaling platform tailored for suppressive activity (46). In addition, semaphorin 4A (Sema4A) secreted by plasmacytoid dendritic cells engages neuropilin-1 (Nrp1) on Tregs, modulating their stability and survival through the PTEN-Akt-Foxo axis (47). Owing to their structural stability, streamlined signaling circuitry, and metabolic adaptability, Tregs function as highly efficient immune brakes within the tumor microenvironment. Targeting Treg synapse formation or key molecules such as Nrp1 and CTLA-4 may therefore disrupt tumor-associated immune tolerance and provide new avenues for combination immunotherapy.

#### 3.2.3 Non-canonical ISs of γδ T cells and CAR-T cells

Although γδ T cells are natural lymphocytes and CAR-T cells are engineered products, both form non-classical ISs that deviate from the canonical bull’s-eye architecture. Vγ9Vδ2 T cells can form bull’s-eye-shaped cytotoxic ISs similar to those of CTLs, but their antigen recognition is MHC-independent (**Table 1**). This subset directly senses phosphoantigens (e.g., isopentenyl pyrophosphate, IPP) that accumulate on the tumor cell surface as a result of metabolic dysregulation, a process mediated by butyrophilin family members BTN3A1 and BTN2A1 and operating through an “inside-out” signaling mode. Binding occurs between the CDR2/CDR3 regions of the Vγ9 chain within the γ9δ2 TCR and BTN2A1; subsequently, CD277 (BTN3A) recruits phosphoantigens to the IS in a manner dependent on pAg, where they are anchored through a RhoB-mediated retention mechanism, providing the necessary conditions for costimulatory signaling. This non-classical recognition mode enables γδ T cells to exert cytotoxic function even against MHC-downregulated tumors, underscoring their unique clinical value (38).

In contrast to the classical TCR-mediated IS, CAR-T cells initiate a structurally distinct alternative IS upon recognition of cognate antigen: Lck exhibits an irregular multifocal distribution, actin rings are diminutive, and LFA-1 is diffusely arranged (35). CAR-T cell signaling is stronger, faster, but shorter in duration than TCR-mediated signaling (48); yet CAR-T cells recruit cytotoxic granules to the IS more rapidly and detach from target cells more quickly after killing. Rab11, through interaction with Rab11 family-interacting protein 3 (FIP3) and the dynein–microtubule system, regulates the directional transport of Lck to the IS (49). Notably, loss of CD58, a CD2 ligand on tumor cells, leads to CAR-T cell dysfunction, providing a new explanation for CAR-T resistance in solid tumors (33, 50). Therefore, engineering strategies to restore the CD2–CD58 axis or optimize the spatiotemporal dynamics of CAR signaling represent potential approaches to improve CAR-T efficacy in solid tumors.

#### 3.2.4 NK cell lytic IS: dynamic kinase regulation and DOCK8-dependent cytoskeletal remodeling

The IS of NK cells differs fundamentally from the stable synapse of T cells. Signal integration in the NK cell IS depends on the dynamic balance of kinase activities, including Src homology region 2 domain-containing phosphatase-1 (SHP-1) and ZAP-70, rather than on fixed costimulatory modules, which endows NK cells with the ability to rapidly recognize and respond to target cells within the complex tumor microenvironment (51). NK cells recognize ecto-calreticulin (ecto-CRT) upregulated on the surface of ER-stressed tumor cells through the activating receptor natural cytotoxicity triggering receptor 1 (NKp46), triggering MHC-unrestricted direct killing (52). This mechanism is particularly important for eliminating tumor cells with downregulated MHC I.

The structural stability of the NK cell IS is highly dependent on the dedicator of cytokinesis 8 (DOCK8)–Rac signaling axis. DOCK8 recruits the small GTPase Ras-related C3 botulinum toxin substrate 1 (Rac1) to promote actin reorganization and maintain synaptic membrane integrity (53). Hypoxia or the accumulation of metabolic byproducts in the tumor microenvironment may induce the loss of synaptic membrane protrusions, leading to reduced cytotoxicity. Moreover, engagement of NKR-P1 (CD161) with lectin-like transcript 1 (LLT1) delivers inhibitory signals (54), while excessive activation of SHP-1 can disrupt actin cytoskeletal reorganization and induce tolerance toward tumor target cells (55). Upon IS formation with tumor cells, localized energy consumption also triggers mitochondrial depolarization in NK cells, further compromising their sustained killing capacity. Therefore, genetic engineering to enhance DOCK8 expression or blockade of inhibitory receptors holds promise for restoring NK cell synaptic efficacy in solid tumors.

#### 3.2.5 B cell antigen-extraction IS: BCR enrichment and the CD20–CD70 axis

The B cell IS is highly dynamic and relatively short-lived, with its core function being the mechanical force-driven extraction and internalization of surface-tethered antigens (56). The synaptic periphery is enriched with F-actin networks, while the central region undergoes MTOC polarization, supporting antigen processing and presentation. Within the tumor microenvironment, B cells recognize tumor-specific antigens (TSAs) or tumor-associated antigens (TAAs) via the B cell receptor (BCR), forming an IS that enriches BCRs and excludes inhibitory receptors such as CD45 (56). Membrane-bound antigens are acquired through a “pinch-off” mechanism involving membrane vesicles rather than proteolytic cleavage, and are subsequently processed into peptide–MHC class II complexes (57). This process is a prerequisite for the activation of T follicular helper (Tfh) cells, which deliver critical costimulatory signals such as CD40L and IL-21 to promote B cell affinity maturation and class switch recombination, ultimately driving the differentiation of antitumor plasma cells (58).

The IS function of B cell is regulated by Bruton’s tyrosine kinase (Btk) and the SNARE protein vesicle-associated membrane protein 7 (Vamp-7) (57). Btk lies at the core of the BCR signaling pathway; Btk inhibitors (e.g., ibrutinib, zanubrutinib) are effective in B cell malignancies but may also impair normal B cell antigen presentation capacity (59, 60). Vamp-7 mediates localized lysosomal exocytosis at the IS, which is indispensable for efficient antigen extraction and presentation (61). Furthermore, CD20 may act as a membrane organizer, forming a complex with CD70 that is recruited to the IS center and establishing a trans-synaptic signaling axis with CD27 on the T cell surface; CD20 deficiency leads to reduced CD70 recruitment and IS structural defects (62). These findings suggest that targeting the B cell IS may simultaneously affect antitumor humoral immunity and T cell responses, requiring careful consideration in therapeutic design (63).

#### 3.2.6 Dendritic cell antigen-presentation IS: FcR pathways and cross-presentation

Dendritic cells (DCs) are central to the initiation of both T cell and B cell responses, and their IS functions involve two complementary Fc receptor (FcR) pathways (62). Activating FcRs primarily generate T cell epitopes and initiate T cell activation, while independently promoting B cell proliferation and antibody secretion. In contrast, the inhibitory receptor Fc gamma receptor IIB (FcγRIIB) preserves conformationally intact B cell epitopes and delivers them to B cells via synaptic vesicle transfer, thereby enhancing B–T cell collaboration (64). This dual-pathway mechanism ensures the efficiency and breadth of antitumor humoral immunity.

Migratory DCs (e.g., CD103^+^ conventional dendritic cell type 1, cDC1) capture antigens in the tumor microenvironment and migrate to draining lymph nodes in response to C-C chemokine receptor type 7 (CCR7) ligands (CCL19/CCL21), where they cooperate with resident DCs to form ISs and transfer processed antigens to CD8^+^ T cells (65). Antigens can persist in DCs for more than 48 hours (57, 66), ensuring effective lymphocyte priming in secondary lymphoid organs (36). Productive IS formation upregulates MHC I expression, enhances CD40 signaling, and induces lipid peroxidation in DCs, thereby providing sufficient costimulation and antigen presentation capacity to prevent T cell anergy (67, 68). In addition, exosomes secreted by DCs, T cells, and B cells can carry MHC molecules, miRNAs (29), and pro-apoptotic factors, acting through the synaptic cleft on target cells to further amplify antitumor immunity (40). These processes rely on the integration of biochemical signals and are also regulated by the physical properties of the tumor microenvironment (e.g., matrix stiffness (69)), providing a theoretical basis for DC-based vaccine design and IS functional optimization (70).

In summary, different immune cells execute antitumor functions through ISs with distinct characteristics. Their structural plasticity, signaling networks, and mechanical sensing capacities profoundly influence the trajectory of antitumor immune responses. Interventions targeting these cell type-specific synaptic mechanisms hold considerable promise for precisely enhancing antitumor immunity.

## 4 Dysregulation of the immune synapse in the tumor microenvironment

### 4.1 Molecular dysregulation of the immune synapse

IS dysfunction within the TME is a multi-layered and multifactorial process involving aberrant membrane molecule expression, disrupted signaling cascades, and genetic or epigenetic alterations in cytoskeletal and polarity regulators. Collectively, these pathological changes lead to failed IS assembly or impaired function, preventing immune cells from effectively recognizing and eliminating tumor cells and ultimately facilitating immune escape. Elucidating these molecular mechanisms is therefore of considerable clinical importance for developing strategies to restore IS integrity and enhance the efficacy of immunotherapy.

#### 4.1.1 Membrane molecule aberrations: adhesion molecule downregulation and inhibitory ligand interference

##### 4.1.1.1 LFA-1/ICAM-1 adhesion axis dysfunction

LFA-1 (αLβ2 integrin) serves as an anchoring adhesion molecule essential for stabilizing IS architecture. Upon antigen recognition, inside-out signals induce the transition of LFA-1 from a low-affinity to a high-affinity conformation, enabling tight binding to its ligand ICAM-1. This process provides mechanical support for the synapse while also coupling with PI3Kδ signaling to promote MTOC polarization, thereby directing the secretion of cytotoxic granules along microtubules into the synaptic cleft (14). However, tumor cells frequently downregulate ICAM-1 expression to undermine LFA-1-mediated adhesive stability. In chronic lymphocytic leukemia, T cells exhibit a marked functional deficiency of LFA-1, resulting in impaired IS assembly, which can be reversed by the immunomodulatory agent lenalidomide through restoration of RhoA activity (71). Furthermore, tumor cells can silence ICAM-1 expression via UHRF1–DNMT1-mediated DNA methylation, further weakening LFA-1/ICAM-1-dependent synaptic anchoring (72) (**Figure 3**). Magnesium supplementation has been shown to improve the high-affinity conformation of LFA-1, suggesting that correcting ionic imbalances within the TME may help restore IS function (73). Recent evidence also indicates that LFA-1 contributes to IS regulation by sensing intercellular mechanical tension; mechanical softening of tumor cells may reduce this tension, thereby facilitating immune evasion (69).

**Figure 3:**
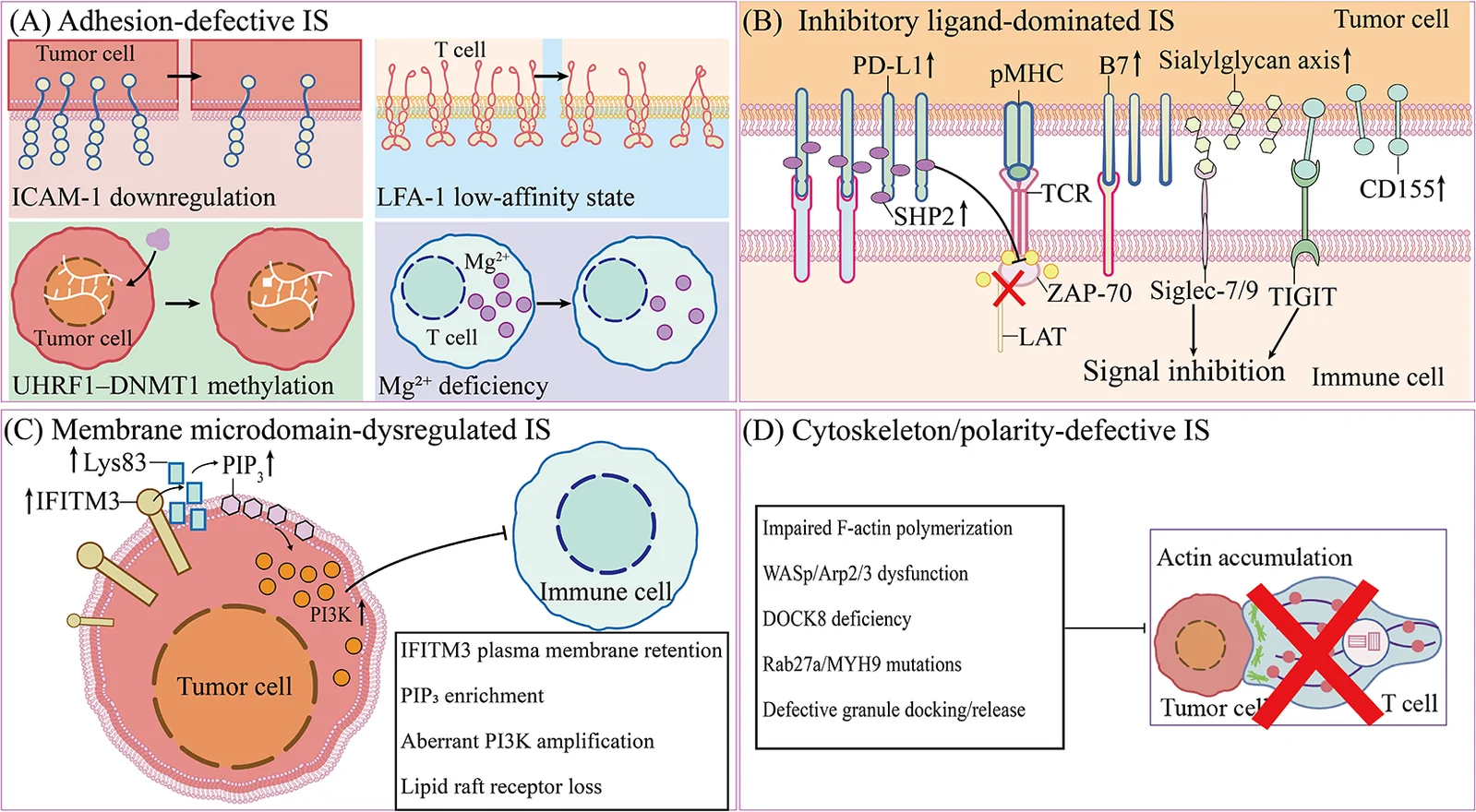
Schematic representation of the multifaceted molecular mechanisms driving immune synapse defects in tumor immunity. This diagram summarizes four distinct pathological categories that disrupt T-cell immune synapse (IS) formation and impair signal transduction within the tumor microenvironment. **(A)** Adhesion-defective IS: UHRF1–DNMT1-mediated epigenetic methylation downregulates ICAM-1 on tumor cells, whereas microenvironmental Mg^2+^ deficiency drives LFA-1 into a low-affinity conformation on T cells. These alterations synergistically abrogate the initial physical intercellular contact required for IS assembly. **(B)** Inhibitory ligand-dominated IS: Overexpression of inhibitory ligands on tumor cells, including PD-L1, B7, the sialylglycan axis, and CD155, engages their respective receptors PD-1, Siglec-7/9, and TIGIT on T cells. This engagement facilitates the recruitment and upregulation of SHP2 phosphatase, which subsequently dephosphorylates and blocks the activation of ZAP-70 and LAT within the TCR complex, leading to potent signal inhibition. **(C)** Membrane microdomain-dysregulated IS: Aberrant upregulation and Lys83-ubiquitination-mediated plasma membrane retention of IFITM3, combined with PIP_3_ enrichment, aberrant PI3K amplification, and lipid raft receptor loss, disrupt the spatial organization of membrane lipid microdomains. This impedes the competent assembly and signal transduction of immune receptor complexes. **(D)** Cytoskeleton/polarity-defective IS: Impaired F-actin polymerization, WASp/Arp2/3 complex dysfunction, DOCK8 deficiency, and Rab27a/MYH9 mutations collectively prevent centrosome polarization toward the IS and fail to support cytolytic granule docking and exocytosis. Consequently, the directional cytolytic capacity of effector T cells is severely impaired in these settings.

Interference by inhibitory ligands. Tumor cells commonly upregulate multiple immunoregulatory ligands, including the B7 family members PD-L1 (CD274) and B7-H3 (CD276), as well as the immunoglobulin superfamily member CD200 (74). These molecules engage inhibitory or competitive receptors on T cells within the IS, disrupting proximal TCR signal transduction. Taking the PD-1/PD-L1 axis as an example, upon PD-L1 binding to PD-1 at the IS, the intracellular domain of PD-1 recruits SHP2 phosphatase, which directly dephosphorylates key TCR signaling molecules such as ZAP-70 and LAT, thereby suppressing TCR microcluster aggregation and signal output (5). In addition, sialylated glycans upregulated on tumor cells are recognized by the inhibitory receptors Siglec-7 and Siglec-9 on NK cells, leading to reduced IS stability and impaired NK cell cytotoxicity; blockade of these receptors can restore antitumor activity (75). Aberrant overexpression of fragile X messenger ribonucleoprotein protein (FMRP) in tumor cells has also been shown to interfere with TCR signaling and inhibit T cell activation and IS formation (76). In the context of CAR-T cell therapy, tumor cell N-glycan modifications mediated by mannosylglycoprotein β-1,6-N-acetylglucosaminyltransferase 5 (MGAT5) disrupt proper IS formation, reducing transcriptional activation, cytokine production, and cytotoxicity, thereby diminishing CAR-T cell killing efficiency (77).

IFITM3 and aberrant PI3K signal amplification. Interferon-induced transmembrane protein 3 (IFITM3) functions as a phosphatidylinositol-3,4,5-trisphosphate (PIP_3_) scaffold protein and a central amplifier of PI3K signaling localized at endosomes (78) (**Figure 3**). In B cell leukemia, oncogenic kinases phosphorylate IFITM3 at Tyr20, causing its persistent retention at the plasma membrane. IFITM3 enriches PIP_3_ via two lysine residues (Lys83 and Lys104) within its intracellular loop, thereby amplifying PI3K signaling. In Ifitm3-deficient B cells, PIP_3_ is markedly depleted from lipid raft microdomains, leading to defective expression of more than 60 lipid raft-associated surface receptors and consequently impairing BCR signaling and cellular adhesion (78). This finding reveals the profound impact of membrane microdomain lipid environments on IS function and provides a rationale for targeting the IFITM3–PI3K axis to restore B cell synaptic competence.

Aberrant epigenetic regulation. Epigenetic alterations within the TME can systematically reprogram IS-related gene expression (79). Tumor cells silence co-stimulatory molecules (e.g., CD40, HLA-A) and adhesion molecules (e.g., ICAM-1) through DNA methyltransferase (DNMT)-mediated hypermethylation, while maintaining immune checkpoint molecules (e.g., PD-L1, HHLA2) in a hypomethylated state, thereby restricting the sustainability of IS signaling (71). Methylation-mediated suppression of interferon regulatory factor 7 (IRF7) may also attenuate the type I interferon network under its control, further limiting T cell cytotoxic function and IS structural integrity (80). Notably, distinct methylation patterns of IS-associated genes between tumor and normal tissues may serve as potential biomarkers for early detection or prediction of immunotherapy response (81).

SLAMF6 and T cell immunoreceptor with immunoglobulin and ITIM domains (TIGIT). SLAMF6 (CD352) serves as a critical adhesion molecule that facilitates tight conjugate formation between T cells and target cells. Within the TME, its expression is markedly suppressed. This downregulation limits the duration of T cell signal integration and transmission, thereby compromising the structural integrity and stability of the IS (82). The TIGIT suppresses IS formation and stability through engagement with its ligands, CD155 and CD112. This ligation event triggers signaling via the intracellular immunoreceptor tyrosine-based inhibitory motif (ITIM) and the immunoglobulin tail tyrosine (ITT) motif of TIGIT. Consequently, downstream signal transduction in NK cells is attenuated (83).

#### 4.1.2 Genetic defects in cytoskeletal and polarity regulators

IS formation is highly dependent on immune cell polarization, including cytoskeletal reorganization, directed transport of intracellular cargo toward the synaptic interface, and precise MTOC positioning. Within the TME, key factors governing these processes are frequently rendered dysfunctional by mutations or aberrant expression, resulting in the failure to properly deliver and release cytotoxic granules.

Defects in Rho GTPase signaling and actin polymerization. The dynamic balance of F-actin polymerization underpins IS structural establishment and maintenance. Rho family GTPases (particularly Rac1 and Cdc42), the Arp2/3 complex, and filamin-A are core regulators of F-actin nucleation and crosslinking (84). RASGRP1 deficiency impairs actin polarization within the IS, at least in part through defective Ras-dependent signaling (85). Mutations in Wiskott–Aldrich syndrome protein (WASp) or its associated WAVE regulatory complex (WRC) disrupt F-actin nucleation and reorganization, leading to defective IS formation and impaired cytotoxic function (86). Similarly, Coronin 1A mutations can disturb T cell homeostasis and IS assembly (87, 88); Vav1 (89, 90), a guanine nucleotide exchange factor for Rac/Rho, when mutated, affects T cell development and IS signal transduction (91).

Dysregulation of the DOCK8–Cdc42 axis. DOCK8 is a specific guanine nucleotide exchange factor for Cdc42, highly expressed in lymphocytes (92, 93). By activating Cdc42, DOCK8 cooperates with WASp to promote actin reorganization, maintaining the integrity of IS membrane protrusions and the directional transport of cytotoxic granules. Patients with DOCK8 deficiency exhibit pronounced IS formation defects and impaired killing capacity in both T cells and NK cells, which is closely associated with weakened tumor immune surveillance (94). In NK cells, DOCK8–Cdc42-dependent actin remodeling is particularly critical for maintaining synaptic membrane integrity; TME factors such as hypoxia or metabolic byproduct accumulation can induce the loss of synaptic membrane protrusions, further exacerbating the consequences of DOCK8 insufficiency (95).

Mutations affecting cytotoxic granule transport and exocytosis. Rab27a is a key small GTPase regulating the docking and fusion of lytic granules with the plasma membrane. Mutations in Rab27a prevent effective anchoring of cytotoxic granules to the synaptic membrane, thereby blocking the release of perforin and granzyme B (96). Non-muscle myosin IIA, encoded by MYH9, facilitates the directional transport of granules along the F-actin network toward the IS through physical interaction with lysosomal granules in NK cells. MYH9 mutations disrupt this process, causing cytotoxic granules to be retained in the cytoplasm (97). Additionally, aberrant expression of lysosome-associated membrane proteins LAMP-1 and LAMP-2 also impedes the release of cytotoxic granules, further weakening IS effector function (98).

Clinical translational implications. The defects in cytoskeletal and polarity regulators described above suggest that restoring these pathways may represent an effective strategy for enhancing antitumor immunity. For example, Rho GTPase agonists or F-actin stabilizers (e.g., jasplakinolide) can reinforce IS scaffolding; genetic engineering to restore DOCK8 (99, 100) or Rab27a (101) expression may improve the killing efficacy of NK cells and CTLs; and modulators targeting MYH9 (102, 103) or LAMP-1/2 may offer additional avenues for restoring IS function. Combination of these approaches with immune checkpoint blockade or adoptive cell therapy holds promise for overcoming immune escape driven by cytoskeletal defects in solid tumors (**Figure 3**).

### 4.2 Cooperative immunosuppressive mechanisms in the tumor microenvironment

The IS serves as the central structural platform for signal integration and effector execution between immune cells and target cells. Its stability and functional integrity depend on the intrinsic molecular machinery of immune cells and are also profoundly influenced by the coordinated actions of metabolic perturbations, soluble mediators, physical barriers, and neural signals within the TME. Through metabolic competition, the release of immunosuppressive factors, matrix remodeling, and neuro-immune crosstalk, the TME systematically undermines IS assembly and effector function, thereby facilitating immune escape. Elucidating these cooperative immunosuppressive mechanisms is of critical importance for designing combination therapeutic strategies that aim to restore IS function by targeting the TME (**Figure 4**).

**Figure 4:**
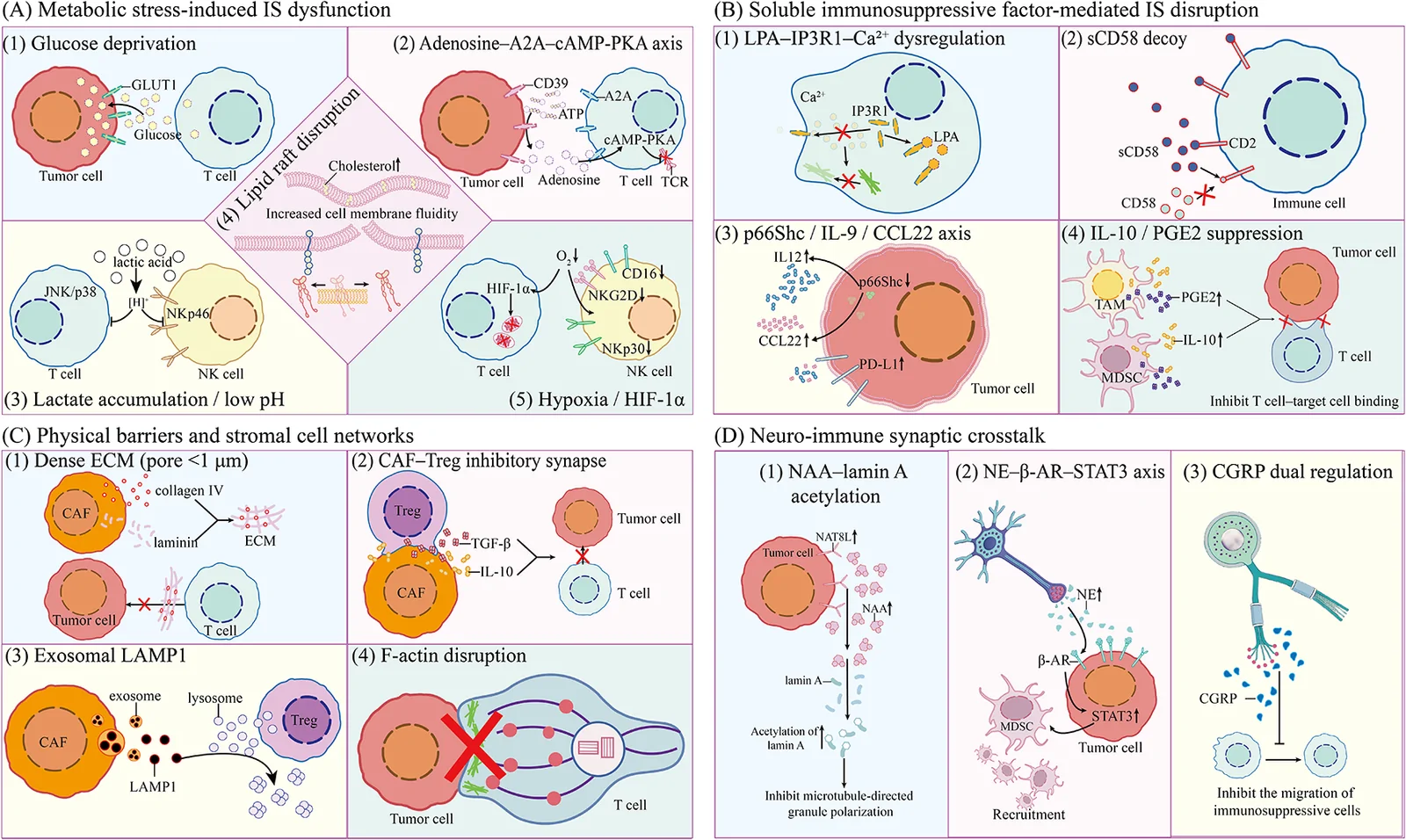
Multilayered mechanistic landscape of immune synapse dysfunction within the tumor microenvironment. This comprehensive schematic delineates four overarching categories of pathological mechanisms driving immune synapse (IS) signal failure and impaired T/NK cell antitumor efficacy in the tumor microenvironment (TME). **(A)** Metabolic stress-induced IS dysfunction: Encompassing various metabolic suppression pathways, including (1) GLUT1-mediated glucose deprivation; (2) adenosine–A2A–cAMP–PKA axis activation via the CD39 ectonucleotidase pathway, which inhibits TCR signaling; (3) lactate accumulation and low pH suppressing NK cell activity via JNK/p38 and NF-κB signaling; (4) lipid raft disruption and increased membrane fluidity interfering with signalosome assembly; and (5) hypoxia/HIF-1α pathways downregulating activating receptors NKG2D and NKp30. **(B)** Soluble immunosuppressive factor-mediated IS disruption: Comprising (1) dysregulation of the LPA–IP3R1–Ca^2+^ axis; (2) soluble CD58 (sCD58) acting as a decoy receptor that blocks the interaction between CD2 on T cells and membrane-bound CD58; (3) tumor cells promoting tumor-associated macrophage (TAM) and regulatory T cell (Treg) infiltration via the p66Shc/IL-9/CCL22 axis; and (4) TAMs and myeloid-derived suppressor cells (MDSCs) secreting IL-10 and PGE2 to directly inhibit T cell–target engagement. **(C)** Physical barriers and stromal cell networks: (1) Dense extracellular matrix (ECM, pore size <1 µm) secreted by cancer-associated fibroblasts (CAFs) acting as a spatial physical barrier to sequester T cells; (2) CAFs forming inhibitory synapses with Tregs, releasing TGF-β and IL-10; (3) CAF-derived exosomal LAMP-1 transferred to Tregs via lysosomal pathways, mediating intercellular communication; and (4) F-actin cytoskeletal disruption impairing the polarization and exocytosis of cytolytic granules. **(D)** Neuro-immune synaptic crosstalk: (1) NAT8L-mediated production of N-acetylaspartate (NAA) causing excessive lamin A acetylation, which inhibits microtubule-directed granule polarization; (2) Norepinephrine (NE) recruiting MDSCs via the β-AR–STAT3 axis; and (3) Calcitonin gene-related peptide (CGRP) exerting dual regulation by inhibiting the migration of immunosuppressive cells.

#### 4.2.1 Metabolic competition and energy depletion

Metabolic dysregulation within the TME represents a major mechanism of IS suppression. By altering local nutrient composition, accumulating metabolic waste products, and hijacking essential energy substrates, tumor cells directly limit the metabolic adaptability and effector polarization of immune cells.

##### 4.2.1.1 Glucose deprivation and adenosine pathway activation

Tumor cells preferentially utilize glycolysis through the Warburg effect, consuming large quantities of glucose and releasing lactate, which leads to marked glucose deprivation within the TME (**Figure 4**). Activated T cells require coordinated metabolic reprogramming involving both glycolysis and oxidative phosphorylation (OXPHOS) to sustain proliferation, survival, and the energetic demands of IS assembly. When T cells are outcompeted for glucose, they fail to achieve the high glycolytic rate necessary for full effector function. This metabolic imbalance disrupts microtubule dynamics and MTOC polarization, impairs the directional transport of cytotoxic granules, and ultimately attenuates IS-mediated killing (104). Concurrently, the adenosine pathway, orchestrated by the ectonucleotidases CD39 and CD73, is markedly activated within the TME. CD39 hydrolyzes extracellular adenosine triphosphate (ATP) to AMP, and CD73 subsequently converts AMP to adenosine. Adenosine engages A2A and A2B receptors on the T cell surface, activating the cAMP–PKA signaling cascade, which suppresses proximal TCR signaling, interferes with TCR microcluster aggregation, and destabilizes IS architecture. Accordingly, targeting the CD39/CD73 axis or blocking adenosine receptors is emerging as an important metabolic intervention to restore IS function (105).

##### 4.2.1.2 Glycosylation-mediated regulation of synaptic receptors

N-glycosylation of the T cell surface glycocalyx, particularly β1,6-GlcNAc branching, directly participates in the modulation of molecular clustering and signal strength at the IS by influencing the spatial conformation and ligand affinity of TCRs, co-stimulatory/co-inhibitory receptors, and cytokine receptors. Studies have shown that deletion of MGAT5 alters TCR clustering and signaling output, indicating a non-linear regulatory effect of glycosylation on IS function (106). The underlying mechanism likely involves altered receptor spatial organization rather than simply enhanced clustering (107). Furthermore, TCR signals themselves coordinately regulate the expression of multiple N-glycan processing enzymes, suggesting that T cell activation and metabolic reprogramming can be integrated at the molecular level through dynamic glycosylation remodeling. Targeting glycosylation pathways, such as with MGAT5 inhibitors, may offer a new means to finely tune IS signal intensity, although the bidirectional effects on T cell activation must be carefully weighed (108).

##### 4.2.1.3 Lactate accumulation and acidic microenvironment

Lactate is one of the most representative metabolic waste products in the TME. High concentrations of lactate are associated with suppressed T/NK function and impaired IS assembly. At the metabolic level, lactate inhibits pyruvate carboxylase (PC) activity, forcing pyruvate into the tricarboxylic acid cycle via pyruvate dehydrogenase (PDH), which reduces succinate secretion and disrupts the autocrine signaling circuit mediated by succinate receptor 1 (SUCNR1) (109). This leads to suppressed mitochondrial OXPHOS and reduced ATP production, weakening microtubule dynamics and MTOC polarization (110). At the signaling level, lactate reduces TCR-triggered phosphorylation of JNK, c-Jun, and p38 (111), inhibits T cell activation, and decreases the secretion of IFN-γ, granzyme B, and TNF-α (112). Moreover, lactate downregulates the expression of the activating receptor NKp46 on NK cells, thereby impairing their IS-dependent killing capacity, while simultaneously promoting Foxp3 expression in Tregs and enhancing their immunosuppressive function (113). Lactate also stabilizes hypoxia-inducible factor 1α (HIF-1α), drives M2 macrophage polarization, and induces epigenetic modifications such as histone lysine lactylation, further consolidating the immunosuppressive milieu (114). Interventions targeting the lactate pathway, such as the MCT1 inhibitor AZD3965 and systemic buffering agents, have shown promise in preclinical models for restoring T cell IS function and enhancing immunotherapy responses (112, 115).

##### 4.2.1.4 Hypoxia and mitochondrial dysfunction

The hypoxic environment within solid tumors directly suppresses mitochondrial biogenesis and OXPHOS, resulting in insufficient energy supply for sustained T cell activation and diminished persistent cytotoxic activity. Activation of hypoxia-inducible factors (HIFs) further reshapes T cell metabolic priorities, enforcing reliance on glycolysis for adaptation to low oxygen tension. However, because tumor cells competitively consume glucose, T cells remain at an energetic disadvantage even within the glycolytic pathway, ultimately exacerbating metabolic exhaustion and impairing long-term IS maintenance. Hypoxia also downregulates the expression of activating receptors on NK cells, including natural killer group 2 member D (NKG2D), natural cytotoxicity triggering receptor 3 (NKp30), and CD16, limiting cytokine production and cytotoxicity (116, 117). In addition, hypoxia induces autophagic activity that selectively degrades connexin 43 (Cx43) (118), disrupting gap junction-mediated communication and IS stability between NK cells and tumor cells (119). On the tumor cell surface, hypoxia upregulates sialylated ligands such as CD59, which engage inhibitory receptors Siglec-7 and Siglec-9 on NK cells, further inhibiting IS formation and reducing the accumulation of granzyme B and F-actin at the synaptic interface (75).

##### 4.2.1.5 Lipid metabolic imbalance and disruption of membrane rafts

Lipid metabolic dysregulation represents another important dimension of IS impairment within the TME. Tumor cells overexpress CD36 to take up free fatty acids, leading to insufficient lipid substrate availability in T cells and a decline in mitochondrial membrane potential, thereby weakening IS energy supply and signal transduction. Concurrently, altered cholesterol metabolism in tumor cells changes membrane fluidity and mechanical stiffness, which inhibits T cell mechanosensing through the integrin-FAK-RhoA axis and attenuates F-actin accumulation and cytotoxic granule release at the IS (69). Lipid rafts serve as critical signaling platforms for IS assembly; their composition and fluidity directly influence the spatial distribution of LFA-1/ICAM-1 and the stability of the pSMAC. In chronic lymphocytic leukemia, T cell cholesterol synthesis pathways are impaired, leading to defective recruitment of key signaling molecules—such as CD45, Lck, LAT, and PKC-θ—to lipid rafts and consequently disrupting the nanoscale molecular organization of the IS (120). Furthermore, cAMP activates the small GTPase Rap1 through EPAC1/PKA signaling to modulate LFA-1 activity and promote the dynamic organization of adhesion molecules within the IS; decreased cAMP levels within the TME directly compromise pSMAC stability and IS formation (121). Targeting lipid metabolism, such as with the ACAT1 inhibitor avasimibe or CD36 antibodies, has shown potential in preclinical studies to enhance IS function and antitumor activity by restoring membrane cholesterol content or fatty acid availability in T cells (121).

#### 4.2.2 Interference by immunosuppressive factors

Multiple soluble immunosuppressive factors within the TME can directly interfere with IS-related signaling or disrupt the adhesive interface, thereby weakening the effector function of T cells and NK cells.

Lysophosphatidic acid (LPA) is present at markedly elevated local and systemic concentrations in various cancer types. LPA impedes the translocation of inositol 1,4,5-trisphosphate receptor type 1 (IP3R1) to the IS, thereby attenuating calcium release from the endoplasmic reticulum and reducing intracellular Ca^2+^ levels. This defect in calcium signaling is associated with impaired activation and target cell recognition in both T cells and NK cells. In addition, LPA activates the RhoA–mDia1 signaling axis, which perturbs the dynamic equilibrium of F-actin polymerization and inhibits TCR-mediated F-actin assembly. LPA also interferes with the detyrosination of microtubules, diminishing their stability and polarization competence. This microtubule dysregulation further hinders the membrane localization and activation of IP3R1, thereby exacerbating the disruption of IS structural integrity. Collectively, LPA-mediated interference with cytoskeletal dynamics impairs the intracellular transport and polarized delivery of perforin and key cytokines, including IFN-γ, culminating in attenuated T cell cytotoxicity (122).

Abnormal accumulation of soluble CD58 (sCD58) within the TME can block CD2–CD58 interactions at the IS, impairing adhesion recognition and signal transduction in T and NK cells, thereby suppressing IS formation (123). In addition, aberrant expression of CXC chemokines indirectly interferes with IS establishment by affecting immune cell migration and localization within the TME (124). Differential cytokine expression profiles among memory T cell subsets—such as high IL-2 production in central memory T cells (TCM) and elevated IFN-γ secretion in effector memory T cells (TEM)—may also influence IS formation and maintenance, suggesting that the functional heterogeneity of memory subsets within the TME warrants further investigation (31).

Tumor-derived exosomes are important long-range immunosuppressive carriers within the TME. Exosomes carrying immune checkpoint molecules such as PD-L1 and CTLA-4 can be delivered to the IS region, where they activate SHP2-dependent phosphorylation cascades that disrupt F-actin polarization and impair the formation of the LFA-1/ICAM-1 adhesion ring, thereby persistently suppressing the recruitment and function of critical IS effector proteins such as LFA-1 and CD107a (LAMP-1) (125). Inhibitory myeloid cells, including tumor-associated macrophages (TAMs) and myeloid-derived suppressor cells (MDSCs), secrete immunosuppressive factors such as IL-10 and prostaglandin E2 (PGE2), which interfere with the physical conjugation between T cells and target cells and undermine the structural foundation required for productive IS assembly. In chronic lymphocytic leukemia, deficiency of the adaptor protein p66Shc promotes the secretion of IL-9 and CCL22, further enhancing PD-1 expression and impeding the formation of functional ISs by CTLs (126).

#### 4.2.3 Physical barriers and stromal cell networks

The physical properties of the TME and stromal cell networks pose direct obstacles to IS establishment. Cancer-associated fibroblasts (CAFs) secrete laminin and collagen IV, generating a dense extracellular matrix meshwork with pore diameters typically below 1 μm. This physical barrier impedes T cell infiltration and restricts direct contact with tumor cells, thereby interfering with IS establishment (127). CAFs can also express immunomodulatory molecules and release immunosuppressive mediators including transforming growth factor beta (TGF-β) and IL-10 via paracrine mechanisms, directly suppressing the cytotoxic capacity of effector T cells (128–130). More critically, CAFs can present tumor-derived antigenic peptides on MHC class II molecules through the autophagy–lysosome pathway, forming a stable CAF–Treg IS network that locally amplifies immunosuppressive signaling and drives functional exhaustion of cytotoxic T lymphocytes (131). Exosomes derived from CAFs carry lysosome-associated membrane protein 1 (LAMP-1), which promotes lysosomal clustering at the Treg synaptic interface and accelerates the release of inhibitory cytokines (132). In preclinical pancreatic cancer models, blockade of CAF autophagy—for example by targeting ATG7—reduces the intratumoral Treg population by approximately 50% and potentiates the efficacy of PD-1 blockade (133). However, in other models, depletion of α-SMA^+^ CAFs (134) leads to more aggressive disease progression and increased intratumoral Treg accumulation, underscoring the context-dependent roles of CAF subsets (132).

Reverse trogocytosis represents a distinct mechanism whereby tumor cells extract critical surface molecules from CAR-T cells at the IS, thereby masking their antigenic identity and rendering CAR-T cells functionally inert (135). Furthermore, direct physical contact between T cells and tumor cells can disrupt F-actin polymerization at the synaptic interface, further compromising the structural foundation essential for productive IS assembly.

#### 4.2.4 Neuro-immune synaptic crosstalk

Recent studies have revealed that the nervous system regulates the tumor immune microenvironment. This neuro-immune synaptic crosstalk—bidirectional communication between neurons and immune cells mediated by neurotransmitters, neuropeptides, and neurotrophic factors—is emerging as a new dimension of IS functional modulation (136). Tumor cells can hijack the neuron-specific metabolite N-acetylaspartate (NAA) by overexpressing N-acetyltransferase 8-like (NAT8L) to synthesize NAA. This induces acetylation of lamin A at lysine 542, disrupting the interaction between lamin A and Sad1/UNC-84 homology 2 (SUN2), thereby inhibiting dynamic IS assembly and microtubule-directed cytotoxic granule polarization. As a result, the killing efficiency of NK cells and CD8^+^ T cells is significantly reduced (137).

The sympathetic and parasympathetic branches of the autonomic nervous system regulate immune cell function through the release of neurotransmitters. Norepinephrine released by sympathetic nerves acts on β-adrenergic receptors on immune cells, activating signal transducer and activator of transcription 3 (STAT3) signaling, which promotes the recruitment and functional maintenance of myeloid-derived suppressor cells and upregulates PD-L1 expression on tumor cells and stromal cells. This establishes a dual neuro-immune checkpoint inhibitory axis that may hinder IS formation and T cell effector function. In contrast, the parasympathetic nervous system releases acetylcholine via the vagus nerve, acting on muscarinic acetylcholine receptor M2 (CHRM2) on dendritic cells and T cells. This inhibits NLR family pyrin domain containing 3 (NLRP3) inflammasome activation and reduces the production of pro-inflammatory cytokines such as IL-1β and TNF-α, diminishes MDSC accumulation, and may optimize IS stability and antitumor immune responses (138). Sensory nerve fibers release calcitonin gene-related peptide (CGRP) and substance P (SP), which bidirectionally regulate IS dynamic assembly by modulating immune cell differentiation and function. CGRP may indirectly promote IS formation by inhibiting the migration of immunosuppressive cells, whereas SP may directly enhance IS killing efficacy by augmenting NK cell degranulation and the directed release of lytic granules (139). Although the precise mechanisms of neuro-immune synaptic crosstalk remain to be fully elucidated, this emerging field offers a novel perspective for restoring IS function through neuromodulation.

### 4.3 Residual positive mechanisms that promote immune synapse formation in the tumor microenvironment

Although the TME is generally immunosuppressive, it still retains a series of signaling networks capable of initiating or sustaining IS assembly. These residual positive mechanisms provide potential footholds for restoring T cell function. Systematically identifying and reinforcing these mechanisms holds significant translational value for reversing T cell exhaustion and overcoming current immunotherapeutic bottlenecks. This section will elaborate on the “positive energy” signals that promote IS formation within the TME from four dimensions: antigen presentation, stromal cell support, cytokine and complement synergy, and chemokine guidance.

#### 4.3.1 Antigen presentation by myeloid cells: cross-dressing and classical cross-presentation

Tumor cells frequently downregulate their own MHC class I molecules to evade recognition by CD8^+^ T cells. However, inflammatory monocytes and DCs within the TME can circumvent this evasion through a non-classical pathway termed “cross-dressing” (140). This process does not rely on internalization and intracellular antigen processing; instead, intact MHC I-peptide complexes on the tumor cell surface are directly captured via intercellular membrane transfer (e.g., trogocytosis or exosome-mediated transfer) and presented wholesale to CD8^+^ T cells, thereby triggering efficient IS assembly and reactivation (141) (**Figure 5**). This mechanism ensures that even when tumor cells lack MHC I expression, CD8^+^ T cells can still be effectively primed through “borrowed” antigen presentation, which is particularly important for countering immune escape driven by MHC I downregulation. A 2022 study further confirmed that in multiple tumor models, cross-dressing—rather than classical cross-presentation—is one of the dominant pathways driving antitumor CD8^+^ T cell responses (142).

**Figure 5:**
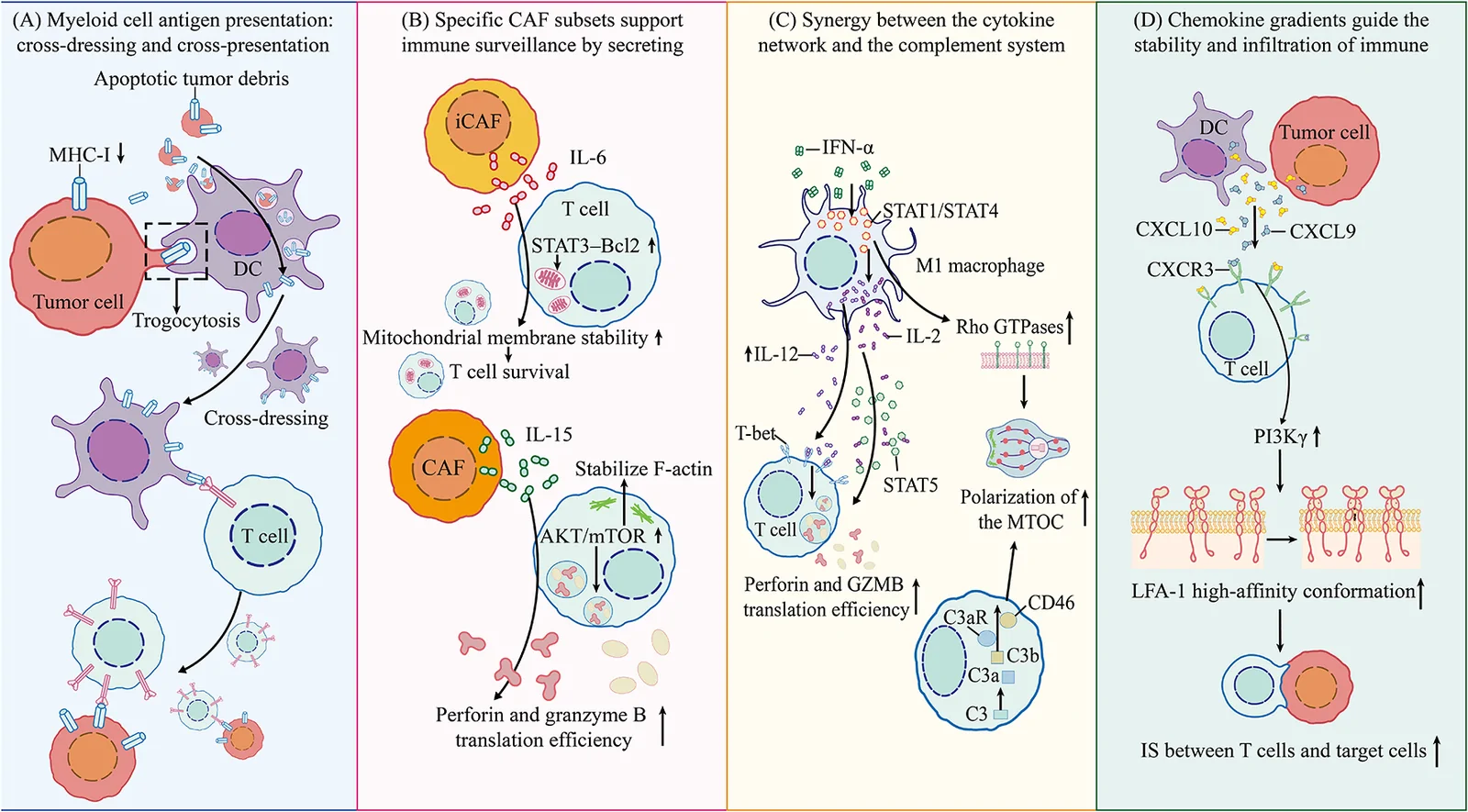
Schematic depiction of multidimensional and synergistic regulatory mechanisms orchestrating T cell immune activation and synapse stabilization within the tumor microenvironment. This schematic delineates how distinct cellular components and signaling networks synergize to promote T cell-mediated antitumor immunity in the tumor microenvironment (TME). **(A)** Myeloid cell antigen presentation via cross-dressing and cross-presentation: Tumor cells with downregulated MHC-I release apoptotic debris, which is subsequently internalized by dendritic cells (DCs). Through cross-presentation and cross-dressing mechanisms, tumor antigens are presented to T cells, thereby initiating and amplifying antitumor T cell responses. **(B)** Specific CAF subsets support immune surveillance by secreting soluble factors: Inflammatory CAFs (iCAFs) secrete IL-6 to activate the STAT3–Bcl2 axis, enhancing mitochondrial membrane stability and promoting T cell survival. Concurrently, CAFs secrete IL-15 to activate the AKT/mTOR pathway, stabilizing F-actin and significantly increasing the translational efficiency of perforin and granzyme B (GZMB). **(C)** Synergy between the cytokine network and the complement system: IFN-α–licensed M1 macrophages secrete IL-12 to upregulate T-bet, enhancing effector molecule translation. Additionally, M1 macrophage-derived IL-2 signals via STAT5 to upregulate Rho GTPases, facilitating microtubule-organizing center (MTOC) polarization. Complement activation yields C3a and C3b fragments, which engage C3aR and CD46 on T cells, respectively, synergistically boosting perforin and GZMB expression. **(D)** Chemokine gradients guide immune infiltration and synaptic stabilization: DC-derived CXCL10 binds to CXCR3 on T cells, activating the PI3Kγ signaling cascade. This induces a high-affinity conformational change in LFA-1, thereby stabilizing and reinforcing the formation of robust immune synapses between T cells and target cells.

Concurrently, classical type 1 dendritic cells (cDC1s) retain the capacity for canonical cross-presentation: they engulf apoptotic tumor cell debris and load exogenous antigens onto their own MHC I molecules through cytosolic pathways, driving the differentiation of naïve CD8^+^ T cells into effector/memory cells and inducing the formation of functional ISs (141). The development and function of cDC1s are highly dependent on the transcription factors Batf3 and IRF8, and their absence markedly impairs antitumor immunity. These two pathways are complementary, underscoring the central role of antigen-presenting cells in tumor immunity—regardless of the presentation route, providing stable and sustained antigenic signals is always the fundamental prerequisite for IS initiation and effector amplification. In terms of clinical translation, vaccine strategies targeting cDC1s (e.g., Flt3L, GM-CSF combined with TLR agonists) and antibody engineering to enhance cross-dressing (e.g., promoting Fc receptor-mediated membrane transfer) are under active exploration (143).

#### 4.3.2 Specific CAF subsets support IS through IL-6/IL-15 secretion

Traditional views have broadly categorized CAFs as immune exclusion barriers, but single-cell multi-omics studies have revealed a high degree of functional heterogeneity among CAFs (144). Based on transcriptional profiles, CAFs can be roughly divided into inflammatory CAFs (iCAFs), myofibroblastic CAFs (myCAFs), and antigen-presenting CAFs (apCAFs), with distinct subsets exerting markedly different effects on the IS. Among them, iCAFs can activate the STAT3–Bcl2 signaling pathway in T cells through paracrine IL-6, enhancing mitochondrial membrane stability and upregulating anti-apoptotic proteins, thereby providing a survival basis for long-term IS maintenance (145). Another CAF subset secretes IL-15, which, by inducing the AKT/mTOR signaling axis, enhances the translational efficiency of perforin and granzyme B while also maintaining the continuous stability of the F-actin network and signaling microclusters within the IS, thereby prolonging the time window of T cell killing (146). IL-15 also promotes the survival and turnover of memory CD8^+^ T cells, which is crucial for long-term immune surveillance (147).

This differential support model of the “CAF subset–cytokine–signaling pathway” overturns the entrenched notion that CAFs have exclusively inhibitory functions. It suggests that precisely modulating the cytokine output of specific CAF subsets may represent a new avenue for improving antitumor IS efficacy. For example, selectively depleting myCAFs or promoting the beneficial functions of iCAFs may enhance IS stability within the TME. Currently, targeting CAF heterogeneity remains challenging, but the continuous enrichment of single-cell atlases offers the possibility of developing subset-specific interventions.

#### 4.3.3 Deep synergy between cytokine networks and the complement system in synaptic function

Cytokines regulate IS maturation and directed secretion. IFN-α/β upregulates perforin and granzyme B expression at the transcriptional level through STAT1/STAT4 cascades (148). More critically, this signaling activates Rho family GTPases (such as Cdc42 and Rac1), inducing the polarization of the MTOC toward the contact interface and ensuring that lytic granules are precisely directed along microtubule tracks to the synaptic cleft (149). IL-12, via the transcription factor T-bet, drives terminal differentiation of CD8^+^ T cells toward an effector phenotype and upregulates effector molecule expression, thereby enhancing the killing output of the IS (150). IL-2, through STAT5 phosphorylation, promotes the pre-assembly of lytic granules within the synapse (such as LYST complex-mediated granule maturation), enabling the IS to achieve a burst-like rapid response upon re-encounter with antigen. These three cytokine-signaling pathways jointly enhance IS killing function across three dimensions: transcription, phenotypic commitment, and granule reserve. Clinically, IL-2/IL-15 superagonists and IFN signaling agonists have entered multiple solid tumor trials aimed at reinforcing IS efficacy in T cells.

The complement system as a “signaling double-edged sword”. Complement components within the TME are not unidirectionally immunosuppressive. Rather, there is a delicate balance. Extracellularly, the C3 cleavage fragment C3d can covalently bind to antigen and, via complement receptor CR2 (CD21), bridge antigen-presenting cells and T cells, exerting a molecular adjuvant effect that promotes IS formation (151). Conversely, the iC3b fragment can tag apoptotic/tumor cells as “self” and, through interaction with CR3 (CD11b/CD18), deliver inhibitory signals that induce T cell tolerance (152). Intracellularly, TCR–CD28 costimulatory signals induce autocrine C3 expression via the LFA-1–JNK pathway; intracellular C3 is cleaved by cathepsin L to generate C3a and C3b. The former acts through C3aR and the latter through CD46, cooperatively driving mTORC1-mediated metabolic reprogramming (glycolysis and oxidative phosphorylation), thereby providing bioenergetic support for Th1 differentiation and sustained CD8^+^ T cell killing (104). Once this dual extracellular–intracellular complement circuit is reactivated, it can locally amplify IS signal strength. Therefore, targeting the C3d/iC3b balance or selectively enhancing intracellular complement activation may become a new strategy for reinvigorating IS function. Currently, combination regimens of complement inhibitors (e.g., C5aR antagonists) with immune checkpoint blockade are being evaluated in preclinical models.

#### 4.3.4 Chemokine gradients guide immune synapse stability and infiltration

The migration of CD8^+^ T cells from the tumor periphery into the core and the formation of stable ISs are highly dependent on local chemokine gradients. C-X-C motif chemokine ligand 9 (CXCL9) and CXCL10 secreted by intratumoral DCs or some tumor cells bind to CXCR3 on the surface of CD8^+^ T cells, triggering downstream PI3Kγ signaling that promotes the transition of LFA-1 from a low-affinity to an extended high-affinity conformation (153). This markedly enhances T cell–target cell adhesion and IS stability. In multiple mouse tumor models, blockade of CXCR3 signaling significantly weakens T cell interstitial infiltration and synaptic duration, whereas local delivery of CXCL9 and CXCL10 can re-recruit T cells to establish functional ISs within the tumor parenchyma (154). Moreover, CXCL9/10 can indirectly enhance antigen presentation and IS initiation by attracting NK cells and cDC1s. Therefore, chemokine gradients are not merely “signposts” that guide migration; they are also spatial signals that directly participate in stabilizing IS architecture (**Figure 5**). Chemokine enhancement strategies that overcome matrix barriers (e.g., intratumoral injection of chemokines, oncolytic viruses carrying CXCL9/10) are expected to synergize with immune checkpoint inhibition, particularly for “cold tumors” with insufficient T cell infiltration.

In summary, the residual positive mechanisms within the TME encompass a multi-layered network ranging from antigen presentation and stromal cell support to cytokine and complement synergy and chemokine guidance. A deeper understanding of these mechanisms, coupled with the rational design of combination therapeutic strategies (e.g., combining cDC1 vaccines, IL-15 agonists, and CXCR3 ligands), holds promise for reigniting IS-mediated antitumor immunity within the immunosuppressive “desert”.

## 5 Therapeutic strategies targeting the immune synapse and clinical translation

The central role of the IS in antitumor immunity makes it an attractive therapeutic target. Intervention strategies aimed at the IS seek to restore or enhance synapse assembly efficiency, signal strength, and effector function at multiple levels, thereby overcoming the immunosuppressive constraints of the TME. This section reviews current IS-directed therapeutic strategies and their clinical translational progress from the perspectives of direct structural repair, immune checkpoint blockade, engineered immune cells, metabolic modulation, cytokine support, and nanoengineering.

### 5.1 Direct repair of immune synapse structure and function

#### 5.1.1 Remodeling the adhesive interface: the LFA-1/ICAM-1 axis and auxiliary adhesion molecules

The physical stability of the IS is highly dependent on high-affinity binding between the integrin LFA-1 and its ligand ICAM-1 (**Table 2**). Epigenetic regulation can directly influence this adhesive axis: in preclinical cell-based studies, treatment of lung cancer cells with low-dose decitabine markedly upregulated ICAM-1 expression, leading to stable aggregation of LFA-1 and the adaptor protein LAT at the contact interface and a substantial increase in the number of functional ISs formed by γδ T cells (155). This finding suggests that restoring adhesion molecule expression through epigenetic agents may represent a feasible strategy for enhancing IS function.

**Table 2:** Representative therapeutic strategies targeting the immune synapse and their clinical development.

Category	Strategy	Agent/platform	Target/mechanism	Key trial/registry	Phase/status	Evidence level	Notes/verification
Adhesion/IS structure	Anti-CLDN18.2 mAb	Zolbetuximab	Stabilizes ALCAM–lipid raft aggregation and IS contact	SPOTLIGHT (NCT03504397); GLOW (NCT03653507)	Phase 3; approved	Approved	FDA Oct 2024; PMDA 2024; NMPA 2025 for CLDN18.2+ HER2− gastric/GEJ
Adhesion/IS structure	LFA-1 allosteric agonist	7HP349 (Alintegimod)	Stabilizes high-affinity LFA-1 conformation; enhances T cell–tumor adhesion	NCT04508179 (Phase 1, completed); NCT06362369 (Phase 1b/2a, ongoing)	Phase 1b/2a	Phase I/II	Fast Track designation for anti-PD-1-resistant melanoma (2022); no efficacy data reported as of Oct 2025
Adhesion/IS structure	ICAM-1-targeted nanoparticle	DOX@PMO-Cy5.5-ICAM	ICAM-1 antibody-conjugated mesoporous silica; local drug delivery at IS	No NCT	Preclinical	Preclinical	Tumor volume reduced ~75% in MDA-MB-231 model
Checkpoint blockade	Anti-LAG-3 + anti-PD-1	Relatlimab + Nivolumab	Blocks LAG-3/MHC-II; restores IS costimulation	RELATIVITY-047 (NCT03470922)	Phase 2/3; approved	Approved	Melanoma; FDA approved 2022
Checkpoint blockade	Anti-CTLA-4 + anti-PD-L1	Tremelimumab + Durvalumab	Blocks CTLA-4/B7 and PD-L1/PD-1 axes	HIMALAYA (NCT03298451)	Phase III; approved	Approved	Hepatocellular carcinoma
Checkpoint blockade	Anti-TIGIT + anti-PD-1/PD-L1	Domvanalimab + Zimberelimab; Tiragolumab + Atezolizumab	Blocks TIGIT–CD155/CD112; restores NK/T-cell IS stability	ARC-10 (NCT04736173); SKYSCRAPER-08 (NCT04540211)	Phase III	Phase III	Verify current endpoint and recruitment status
Checkpoint blockade	Neoadjuvant anti-PD-1 ± anti-CTLA-4/LAG-3	Anti-PD-1 mAb ± anti-CTLA-4 or anti-LAG-3	Relieves inhibitory signals; restores TCR microclusters	NCT04080804	Phase 2 neoadjuvant	Phase II	HNSCC; verify arms
Checkpoint blockade	Anti-PD-L1/TIGIT bispecific	HLX301	Dual PD-L1/TIGIT blockade	CTR20221234	Phase 1/2; actively terminated	Phase I/II (terminated)	Sponsor strategic adjustment; CTR20221234 status
Metabolism	IDO1 inhibitor	Epacadostat; BMS-986205	Blocks IDO–Kyn–AhR axis	ECHO-301/KEYNOTE-252 (Phase 3 negative); NCT02658890	Phase 3 negative	Phase III negative	ECHO-301 failed primary PFS endpoint; not established therapy
Metabolism	Dual A2AR/A2BR antagonist	Etrumadenant (AB928)	Blocks adenosine–cAMP–PKA inhibition	NCT03655711; ARC-7 (NCT04262856)	Phase 1/2	Phase I/II	Verify ARC-7 NCT; mCRPC and NSCLC
Metabolism	Anti-CD39 mAb	TTX-030	Blocks ATP→AMP hydrolysis; reduces adenosine	NCT04306900; NCT03884556	Phase 1/1b	Phase I	Gastric/GEJ; + anti-PD-1 + chemo
Metabolism	A2AR antagonist	CPI-444 (ciforadenant)	Relieves adenosine-mediated T-cell suppression	NCT02655822	Phase 1a/1b	Phase I	Hematologic malignancies
Cytokine	IL-15 superagonist	N-803 (Anktiva; nogapendekin alfa inbakicept)	Sustained JAK/STAT + AKT/mTOR activation; stabilizes F-actin and lytic granule expression	QUILT 3.032	Phase 2/3; approved April 2024	Approved	BCG-unresponsive NMIBC CIS ± papillary tumors; + BCG
Cytokine/APC	CD40 agonist	Mitazalimab	Activates APC costimulation; enhances IS initiation	OPTIMIZE-1 (NCT04888312)	Phase 1b/2	Phase I/II	Metastatic PDAC + mFOLFIRINOX
Cell therapy	BCMA CAR-T	Ciltacabtagene autoleucel (cilta-cel)	BCMA-targeted antitumor IS	CARTITUDE-1 (NCT03548207)	Phase 1b/2; approved 2022	Approved	Multiple myeloma
Cell therapy	BCMA CAR-T	Idecabtagene vicleucel (ide-cel)	BCMA-targeted antitumor IS	KarMMa (NCT03361748)	Phase 2; approved 2021	Approved	Multiple myeloma
Cell therapy	Dual-target CAR-T	CD19-22 CAR-T ± NKTR-255/CD19-22.BB.z-CAR T	Simultaneous CD19/CD22 recognition; reduces antigen escape	NCT03233854	Phase I	Phase I	B-ALL/LBCL
T-cell engager	DLL3/CD3 bispecific	Tarlatamab (Imdelltra)	Artificial IS; T-cell redirection to DLL3+ tumors	DeLLphi-301 (NCT05060016)	Phase 2 registrational; FDA accelerated approval May 2024	Approved	ES-SCLC post-platinum
Nanotechnology	Personalized long-peptide neoantigen vaccine	Peptide neoantigen vaccine	Displays neoantigens; drives TCR clustering	NCT02035956	Phase I	Phase I	Platform = long-peptide, not mRNA; separate from sHDL
Nanotechnology	sHDL neoantigen nanodisc	sHDL nanodisc vaccine	High-density neoantigen display; TCR clustering	No NCT	Preclinical	Preclinical	Do not merge with NCT02035956

In this classification, Adhesion/IS structure refers to strategies that directly target the immune synapse, whereas Checkpoint blockade, Metabolic modulation, Cytokine therapy, Cytokine/APC, Cell therapy, T-cell engager, and Nanotechnology-based therapy refer to therapies that indirectly modulate immune synapse function.

In preclinical anti-PD-1-resistant models, the LFA-1 allosteric agonist 7HP349 stabilized the extended high-affinity conformation of LFA-1, thereby enhancing T cell adhesion and migration within the tumor stroma and increased CD8^+^ T cell infiltration (73). In chronic lymphocytic leukemia, the immunomodulatory agent in preclinical and patient-derived chronic lymphocytic leukemia (CLL) T cell studies, lenalidomide has been reported to restore RhoA activity and reverse tumor-induced defects in T cell LFA-1 signaling and F-actin polymerization, whether this translates into clinical IS repair remains unproven (156). Conversely, timely inhibition of LFA-1 also has therapeutic value: in preclinical colorectal cancer models, the small molecule BIRT377, by blocking the high-affinity conformation of LFA-1, promoted NK cell infiltration (157). Such bidirectional regulation suggests that fine-tuning LFA-1 activity according to cell type and tumor stage may be superior to simple activation or inhibition.

ICAM-1 itself can also serve as a target for drug delivery and immune retention. ICAM-1 antibody-conjugated mesoporous silica nanoparticles reduced tumor volume by 75% in a breast cancer model with low systemic toxicity (158). In a melanoma model, blockade of ICAM-1 homotypic interactions “locked” activated CD8^+^ tumor-infiltrating lymphocytes in situ, indicating that ICAM-1 is a critical molecule for maintaining intratumoral T cell retention (159).

Beyond the LFA-1/ICAM-1 main axis, auxiliary adhesion molecules also contribute to fine-tuning of the IS. CD6 binding to ALCAM/CD166 on antigen-presenting cells stabilizes the IS and promotes T cell activation. In rheumatoid arthritis trials, the low-dose non-depleting anti-CD6 antibody itolizumab showed a favorable safety profile; it modulates this interaction to reduce inflammatory responses without completely abolishing T cell function, and its role in tumor IS modulation remains unvalidated (160). Agrin, a key molecule in neuronal synapses, is also expressed in lymphocytes; blocking its function disrupts IS cap structure formation, suggesting that it may be a new target for stabilizing the IS (161). The Sema4A–Nrp1 axis maintains the immunosuppressive function of Tregs within the TME (47). In preclinical models, blockade with anti-Sema4A or anti-Nrp1 antibodies significantly reduces intratumoral Treg numbers and increases polyfunctional CD8^+^ T cell infiltration, thereby releasing inhibition of the IS without inducing autoimmune side effects (162).

#### 5.1.2 Repairing cytoskeletal and polarization mechanisms

IS effector function requires precise cytoskeletal rearrangement and polarized signal distribution. The tight-junction protein claudin 18 (CLDN18), a member of the claudin family of tetraspan-transmembrane proteins, drives actin-mediated aggregation of ALCAM into lipid rafts, enhancing ALCAM–CD6 heterotypic interactions and thereby stabilizing T cell–tumor cell contact. Anti-CLDN18.2 IgG1 antibodies have shown survival benefits in advanced gastric and pancreatic cancers. CLDN18.2-specific CAR-T cells have also demonstrated preliminary antitumor activity for gastrointestinal cancers (163).

Local enrichment of diacylglycerol (DAG) at the IS membrane is central to signal transduction, and its balance is dynamically maintained by sorting nexin 27 (SNX27) and diacylglycerol kinase ζ (DGKζ). In experimental models, overexpression of SNX27 or the use of the DGKζ inhibitor R59903 both elevate DAG levels, promoting MTOC polarization and directed secretion of cytotoxic granules (164). Combination with the microfilament stabilizer jasplakinolide to enhance F-actin polymerization further consolidates IS structure and supports DAG signal retention. The integrity of the microtubule network is equally important: paclitaxel analogs may influence MTOC polarization, while overexpression of the dynein adaptor p150Glued promotes the convergence of secretory granules toward the MTOC (165). From the perspective of signal integration, engineered phosphorylated LAT proteins enhance SLP76 recruitment, optimizing the coupling efficiency between DAG generation and downstream signaling pathways (164).

Protein kinase C-θ (PKC-θ) is a key node linking TCR signaling to cytoskeletal polarization. In effector T cells, PKC-θ activates the nuclear factor kappa B (NF-κB) pathway and drives IS maturation; in Tregs, PKC-θ promotes IS symmetry breaking through a FoxP3-dependent pathway, weakening IS persistence (166). PKC-θ-deficient T cells retain antiviral activity while mitigating graft-versus-host disease, suggesting that the PKC-θ inhibitor AEB071 may be used in engineered T cell therapies to improve safety (167). In addition, optogenetic tools such as OptoSTIM1 and OptoCRAC enable remote encoding of calcium influx and signal intensity within the IS, providing new means for reversible manipulation of IS function (168).

#### 5.1.3 Overcoming physical barriers: cholesterol depletion and matrix remodeling

The softness of the tumor cell membrane constitutes a form of “mechanical immune checkpoint”—a mechanical barrier that limits T cell mechanosensing and stable IS formation. Depletion of membrane cholesterol using methyl-β-cyclodextrin (MeβCD) increases cancer cell stiffness, which in turn induces more robust F-actin accumulation and mechanical responses in T cells at the IS, significantly enhancing the *in vivo* efficacy of adoptive cell therapy. Correspondingly, inhibition of autophagy in cancer-associated fibroblasts (CAFs) can indirectly reduce the physical resistance to T cell penetration by remodeling the extracellular matrix, clearing spatial obstacles for IS formation (69).

### 5.2 Repair of the IS by immune checkpoint blockade

The essence of immune checkpoint blockade (ICB) therapy is, to a large extent, the repair of the IS signaling platform disrupted by inhibitory signals (80). PD-1 recruits the SHP2 phosphatase to directly dephosphorylate proximal TCR signaling molecules within the IS; anti-PD-1/PD-L1 antibodies relieve this inhibition, restoring ZAP-70 and LAT phosphorylation and microcluster aggregation (3). CTLA-4 removes CD80/CD86 from antigen-presenting cells through trans-endocytosis, depriving CD28 of costimulatory signals; the anti-CTLA-4 antibody ipilimumab reverses this process and rebuilds costimulatory microdomains within the IS (169). Combined blockade of PD-1 and CTLA-4 has become a standard regimen in melanoma and other tumor types. New-generation checkpoint blockade targeting the lymphocyte activation gene 3 (LAG-3)/MHC-II axis, such as relatlimab combined with nivolumab, has been approved for melanoma and has shown progression-free survival benefit in a phase III trial (170); targets such as T cell immunoreceptor with immunoglobulin and ITIM domains (TIGIT) and T cell immunoglobulin and mucin domain-containing protein 3 (TIM-3) are under active clinical exploration (83) (**Table 2**).

Notably, tumor-derived exosomes can carry inhibitory ligands such as PD-L1 to remotely weaken IS function. Strategies targeting exosome biogenesis nodes such as N-acetyltransferase 8-like (NAT8L) are expected to synergize with ICB to systematically reshape the inhibitory signaling network at the synapse (137).

### 5.3 Synaptic remodeling of engineered immune cells

#### 5.3.1 Structural and signaling optimization of the CAR-T cell synapse

Resistance of solid tumors to CAR-T cell therapy largely stems from insufficient IS assembly efficiency and inadequate signaling persistence. To overcome this bottleneck, a series of strategies are being developed to reshape the CAR-T cell synaptic structure at the molecular level (171).

Traditional CAR signaling modules cannot fully mimic the signal integration capacity of the natural TCR. By introducing a CD3ϵ intracellular motif capable of liquid–liquid phase separation (LLPS)—a process in which multivalent biomolecules condense into membraneless compartments—into the CAR, signal condensates form intracellularly and enrich key kinases such as ZAP-70 and LAT. In preclinical studies, this design enables CAR-T cells to form functional ISs even upon stimulation with only a few antigen molecules, with killing efficiency approximately 2–3-fold higher than that of second-generation CARs (172). Concurrently, enhancing auxiliary adhesion and costimulatory signals is equally critical for IS stabilization. In preclinical models, overexpression or exogenous supplementation of CD2 and its tumor cell surface ligand CD58 significantly enhanced initial T cell–target cell adhesion and subsequent IS assembly, and remains effective against tumor cells with low-density CD19 variants (173). Integrating the CD99 intracellular domain into the CAR further strengthens the physical coupling between microtubules and the F-actin network, improving IS dynamic stability and serial killing capacity (174).

Prolonging signal lifespan: costimulatory domain selection and scaffold protein recruitment. With regard to signal persistence, replacing CD28 with the 4-1BB (CD137) costimulatory domain confers superior survival, proliferation, and memory differentiation capacity on CAR-T cells through differential metabolic reprogramming and mitochondrial biogenesis (175). In preclinical models, integrating a PDZ-binding motif (PDZbm) into the intracellular tail of the CAR recruits the Scribble polarity complex upon IS formation. This scaffold reinforces the overall synaptic architecture and, more critically, guides the directional transport of lytic vesicles along microtubule tracks to the synaptic cleft, enabling sustained and efficient release of cytotoxic granules (176). The above designs have demonstrated superior *in vivo* persistence and antitumor activity in solid tumor models including diffuse intrinsic pontine glioma and osteosarcoma (177). Based on the finding that IS structural quality can predict clinical efficacy, enhancing CAR-IS formation through scaffold crosslinking has become a core concept for optimizing product potency.

Synergistic arming: oncolytic viruses and epigenetic modulation. Loading immune activators onto oncolytic virus platforms can further reshape the TME to support the IS (178). The third-generation oncolytic adenovirus Delta-24-RGDOX (DNX-2440) incorporates OX40 ligand into its backbone (179), locally delivering costimulatory signals while lysing tumor cells and blocking IDO–Kyn–AhR-mediated immunosuppression, thereby positively modulating IS function. It is currently in clinical trials for malignant gliomas and other solid tumors (180, 181). In preclinical studies, low-dose decitabine can upregulate CD70 expression on acute myeloid leukemia cells, providing stronger *in situ* costimulatory signals for CD70-targeted CAR-T cells and significantly enhancing their proliferation and sustained killing activity (182).

Although CAR-NK cells possess MHC-independent rapid killing capacity, their IS stability is generally inferior to that of T cells. Integration of PDZbm into the cytoplasmic tail of the CAR-NK construct similarly promotes Scribble recruitment to the synapse, enhances pZAP-70 signal intensity, and improves lysosomal polarization efficiency, resulting in significantly prolonged survival in lung cancer and osteosarcoma models (183). Concurrently, overexpression of the activating receptor NKG2D or high-affinity CD16, combined with genetic ablation of the lactate transporter MCT4 or the adenosine receptor A2AR, can systematically enhance NK cell IS efficacy from both recognition initiation and metabolic resistance dimensions (184). Furthermore, TIGIT-knockout CAR-NK cells have shown significant antitumor effects and prolonged survival in mouse models (83) (**Table 2**).

Bispecific antibodies and T cell engagers: constructing artificial ISs (177). T cell engagers (TCEs) simultaneously bind tumor surface antigens and the CD3/TCR complex on T cells, forcibly forming a non-classical artificial IS that bypasses the constraints of natural TCR–antigen presentation. The first-generation bispecific T-cell engager (BiTE) molecule blinatumomab (CD19×CD3) (185) has been approved for B cell acute lymphoblastic leukemia, validating the clinical feasibility of this pathway (15). Anti-CD3×HER2 bispecific antibodies have been shown in breast cancer clinical trials to increase the density of TCR microclusters within the IS, significantly improving T cell antigen recognition and activation capacity. New-generation TCEs, through optimization of linker length and molecular geometry, promote target antigen multimerization and physically exclude the CD45 phosphatase from the IS, thereby greatly reducing the TCR activation threshold. For example, an anti-Fc receptor-like 5 (FcRH5)/CD3 T cell-dependent bispecific antibody efficiently kills myeloma cells at picomolar concentrations (186). The DLL3-targeting TCE tarlatamab has entered phase III combination immunotherapy trials for small cell lung cancer and has demonstrated durable killing potential (187). The delta-like ligand 3 (DLL3)-targeting TCE tarlatamab has entered phase III combination immunotherapy trials for small cell lung cancer and has demonstrated durable killing potential (188). Arming oncolytic viruses with bispecific linkers, such as PD-L1/CD3 linker-modified herpes simplex virus type 1 (HSV-1), can achieve simultaneous checkpoint blockade and CD3 bridging at the tumor site, forming a “pseudo-synapse” for local synergistic immune activation (189). In the face of antigen heterogeneity in solid tumors, tandem CAR-T cells targeting both human epidermal growth factor receptor 2 (HER2) and interleukin-13 receptor subunit alpha 2 (IL-13Rα2) enhance F-actin accumulation and MTOC polarization within the IS through multi-antigen recognition, effectively strengthening synaptic intensity and preventing antigen escape (190). Moreover, bispecific IgG constructs targeting ICAM/platelet endothelial cell adhesion molecule (PECAM) can significantly prolong antibody residence time in the lung, providing a tissue-specific strategy for promoting durable IS formation (8).

Engineering auxiliary immune cells to support IS stability. Beyond direct modification of effector lymphocytes, engineering auxiliary immune cells can indirectly reinforce IS function. B cells activated by CD40 agonists or interferon gamma (IFNγ) can overexpress interleukin-15 receptor alpha (IL-15Rα), thereby enhancing the IS stability of CD8^+^ T cells. In glioblastoma clinical trials, patients treated with engineered B cells (e.g., BVax) exhibited significantly increased proportions of activated CD8^+^ T cells (CD69^+^) (191). Similarly, clustered regularly interspaced short palindromic repeats (CRISPR)/Cas9-mediated gene editing or cytokine stimulation (e.g., IFNγ, GM-CSF) can reprogram immunosuppressive M2 macrophages into pro-inflammatory M1 macrophages, enhancing their capacity to phagocytose tumor cells and present antigens, and to activate T cell ISs through the secretion of IL-12. Furthermore, blockade of “don’t eat me” signals such as CD47, or engineering macrophages to directly secrete PD-L1 blockers, can relieve inhibition of the T cell IS, establishing a positive feedback loop that amplifies antitumor immunity (192). Advances in optimized genome engineering of primary human B cells via CRISPR/Cas9 have opened broad applications in basic research, antibody production, and cell therapy (193).

Collectively, these engineering strategies—ranging from direct molecular optimization of the CAR-T cell synapse to the construction of artificial synapses with bispecific antibodies and the recruitment of auxiliary immune cells—converge on a common goal: restoring the structural and functional integrity of the IS to overcome the barriers imposed by solid tumors.

#### 5.3.2 Synaptic enhancement of CAR-NK and TCR-T cells

NK cells possess unique potential for solid tumor therapy due to their MHC-independent rapid killing, but their IS stability is generally inferior to that of T cells (194). Integrating PDZbm into the cytoplasmic tail of the CAR-NK construct similarly promotes Scribble recruitment to the synapse, enhances phosphorylated ZAP-70 (pZAP-70) signal intensity and lysosomal polarization efficiency, and significantly prolongs survival in lung cancer and osteosarcoma models (195). Concurrently, overexpression of the activating receptor natural killer group 2 member D (NKG2D) or high-affinity CD16, combined with genetic ablation of the lactate transporter monocarboxylate transporter 4 (MCT4) or the adenosine A2A receptor (A2AR), can systematically enhance NK cell IS efficacy from both recognition initiation and metabolic resistance dimensions (196). TIGIT-knockout CAR-NK cells have shown significant antitumor effects and prolonged survival in mouse models. Allogeneic GD2-CAR-T cells (e.g., ALLO_GD2-CART01) as universal products also provide a new treatment option for high-risk neuroblastoma patients who have failed autologous products (197).

For TCR-T cells, genetic overexpression of LFA-1 or ICAM-1 can directly reinforce the adhesive foundation with target cells in preclinical studies. Furthermore, the use of small-molecule agonists such as 7HP349 to stabilize the high-affinity conformation of LFA-1, or knockout of negative regulators such as kindlin-2, can significantly optimize integrin function and accelerate IS maturation and stability.

#### 5.3.3 Bispecific antibodies and T cell engagers: constructing artificial immune synapses

T cell engagers (TCEs) can simultaneously bind tumor surface antigens and the CD3/TCR complex on T cells, forcibly forming a non-classical artificial IS between the two cells and thereby bypassing the constraints of natural TCR–antigen presentation. The first-generation BiTE molecule CD19×CD3 has been approved for B cell acute lymphoblastic leukemia, validating the clinical feasibility of this pathway (198). Anti-CD3×HER2 bispecific antibodies have been shown in breast cancer clinical trials to increase the density of TCR microclusters within the IS, significantly improving T cell antigen recognition and activation capacity.

New-generation TCEs, through optimization of linker length and molecular geometry, promote target antigen multimerization and physically exclude the CD45 phosphatase from the IS, greatly reducing the TCR activation threshold. In preclinical studies, an anti-FcRH5/CD3 T cell-dependent bispecific antibody efficiently kills myeloma cells at picomolar concentrations (186). The DLL3-targeting TCE tarlatamab has entered phase III combination immunotherapy trials for small cell lung cancer, showing durable killing potential (71). Furthermore, the endogenous secreted bispecific antibody platform (STAb) sustains long-term artificial IS stimulation by continuously producing TCEs *in vivo*, addressing the short half-life of conventional bispecific antibodies (188).

In preclinical models, arming oncolytic viruses with bispecific linkers, such as PD-L1/CD3 linker-modified HSV-1, can simultaneously achieve checkpoint blockade and CD3 bridging at the tumor site, forming a “pseudo-synapse” for local synergistic immune activation (199). In the face of antigen heterogeneity in solid tumors, tandem CAR-T cells (e.g., targeting both HER2 and IL-13Rα2) enhance F-actin accumulation and MTOC polarization within the IS through multi-antigen recognition, effectively strengthening synaptic intensity and preventing antigen escape (200). Moreover, bispecific IgG constructs targeting ICAM/PECAM can significantly prolong antibody residence time in the lung, providing a tissue-specific strategy for promoting durable IS formation (201).

### 5.4 Metabolic modulation strategies: optimizing the IS signaling environment and energy supply

Metabolic pathway dysregulation is a major driver of IS functional suppression in the TME; intervening in these pathways can effectively restore synaptic efficacy in preclinical and early clinical studies.

#### 5.4.1 Adenosinergic axis and sialic acid receptors

The CD39/CD73 axis is the principal pathway for extracellular adenosine generation (202). In preclinical and early clinical studies, CD73 inhibitors such as oleclumab block adenosine accumulation and restore TCR signaling (105). The dual A2A/A2B adenosine receptor antagonist etrumadenant relieves adenosine-mediated suppression of calcium flux, extracellular signal-regulated kinase (ERK) pathway activation, and cytokine secretion in CAR-T cells, and has shown preliminary efficacy in combination with chemotherapy and PD-1 antibodies in non-small cell lung cancer (203). In addition, Siglec-7/-9 are immunosuppressive sialic acid receptors on the surface of NK cells and T cells. In preclinical models, polymeric nanoparticles or blocking antibodies (e.g., targeting Siglec-9) (204) that interfere with their binding to tumor ligands significantly enhance IS formation between NK cells and tumor cells, increasing cytotoxicity several-fold and augmenting CD8^+^ T cell activation in prostate cancer models (205).

#### 5.4.2 Lactate and the acidic microenvironment

Lactate produced in large quantities by tumor glycolysis is associated with impairs the adhesive stability of LFA-1/ICAM-1 (112). The monocarboxylate transporter 1 (MCT1) inhibitor AZD3965 and systemic buffering agents such as bicarbonate reduce microenvironment acidity and significantly improve tight contact between T cells and tumor cells (113). In the metabolic branch, pyruvate dehydrogenase (PDH) activators that restore mitochondrial pyruvate oxidation may modulate succinate signaling and enhance T cell killing function and IFNγ production (109).

#### 5.4.3 Lipid metabolism and DAG membrane dynamics

Plasma membrane cholesterol content directly influences the clustering efficiency of TCR nanoclusters. The acetyl-CoA acetyltransferase 1 (ACAT1) inhibitor avasimibe blocks esterification of free cholesterol, increasing membrane cholesterol levels and significantly enhancing TCR microcluster formation and IS signal strength in CD8^+^ T cells (17). For CAR-T cells, activation of the mechanistic target of rapamycin (mTOR)–sterol regulatory element-binding protein 2 (SREBP2)–3-hydroxy-3-methylglutaryl-CoA synthase 1 (HMGCS1) pathway by glutamine to promote *de novo* cholesterol synthesis is critical for maintaining mitochondrial fitness, membrane stability, and durable IS function (206). In preclinical models, blocking CD36-mediated fatty acid sequestration on tumor cells restores fatty acid oxidation and mitochondrial membrane potential in T cells, safeguarding the energy demands of the IS (207).

DAG accumulates at the IS and is distributed along with membrane specialization. By inducing negative membrane curvature, DAG promotes outward budding (ectocytosis) to form DAG-rich extracellular vesicles, facilitating rapid CTL detachment from target cells and supporting serial killing. In experimental models, enhancing phospholipase C gamma (PLCγ) activity or inhibiting DAG-degrading enzymes increased DAG generation and promotes ectocytosis efficiency; delivering DAG analogs via liposomes or nanoparticles can locally enhance membrane curvature at the IS (208). Furthermore, modulating phosphoinositide 3-kinase (PI3K) and phospholipase C (PLC) activities to optimize phospholipid distribution at the IS, using DHHC enzyme activators or inhibitors to regulate membrane localization and function of key proteins such as Orai1 and Lck, and designing lipid probes or synthetic lipids to directly manipulate IS membrane composition all provide new tools for precisely intervening in the IS lipid environment (209).

### 5.5 Synergistic support from cytokines and immunomodulators

The IL-15 superagonist complex N-803, through sustained activation of Janus kinase (JAK)/signal transducer and activator of transcription (STAT) and AKT/mTOR pathways, maintains the F-actin network within the IS while upregulating perforin and granzyme B expression, stabilizing the IS from both structural and functional dimensions. It has been approved in combination with bacillus Calmette-Guérin (BCG) for BCG-unresponsive non-muscle invasive bladder cancer (147, 210), this approval does not directly prove IS-related causality. IL-27, through the STAT1/STAT3 pathway, promotes lysosomal transport and cytotoxic molecule expression; in combination with PD-L1 inhibitors, it significantly improves complete response rates in lung cancer models (211). In preclinical models, fourth-generation armored CAR-T cells (TRUCKs, T cells redirected for universal cytokine killing) can locally and continuously secrete pro-inflammatory cytokines such as IL-12 or IL-15, directly counteracting the destructive effects of TGF-β and IL-10 within the TME on the IS (212). To control the accompanying cytokine release syndrome (CRS), selective blockade of IL-6 trans-signaling with nanobodies such as ALX-0061 can mitigate the inflammatory storm without compromising IS function (196). Furthermore, upregulation of thioredoxin-1 (TRX1) expression enables CAR-T cells to maintain IS formation capacity and sustained proliferative and killing properties in the highly oxidative TME (190). Engineered B cells activated by CD40 agonists or IFNγ can overexpress IL-15Rα, enhancing CD8^+^ T cell IS stability; in glioblastoma clinical trials, a significant increase in CD8^+^ T cell activation markers was observed in patients treated with engineered B cells (190).

### 5.6 Nanoengineering and *in situ* vaccines: reshaping the microenvironment for IS initiation

Synthetic high-density lipoprotein (sHDL) nanodiscs can display tumor neoantigen peptides in a repetitive array, multivalently binding and driving TCR clustering to efficiently induce IS assembly, and have entered clinical trials as personalized neoantigen vaccines (213) (**Table 2**). In preclinical studies, Fe_3_O_4_ magnetic nanoparticles can deliver CpG oligodeoxynucleotides to target dendritic cells, enhancing their maturation through Toll-like receptor 9 (TLR9) activation and thereby improving IS formation efficiency between DCs and T cells (214). Postoperative implantation of reactive oxygen species (ROS)-responsive nanoarray gels can trigger gasdermin E (GSDME)-dependent pyroptosis in tumor cells, releasing large quantities of damage-associated molecular patterns and tumor antigens. This process strongly recruits and activates antigen-presenting cells while simultaneously blocking PD-L1 expression, thereby providing effector T cells with an ideal microenvironment of released inhibition and enhanced IS initiation, and significantly reducing metastasis in models (215). In preclinical studies, engineered exosomes delivering miR-124 or miR-34a can reprogram M2 macrophages toward an M1 phenotype, indirectly supporting IS stability by improving local antigen presentation efficacy (216).

## 6 Advanced technologies and dynamic monitoring in immune synapse research

The nanoscale architecture and rapid dynamics of the IS require advanced imaging and analytical tools. Super-resolution microscopy, live-cell tracking, *in situ* interaction mapping, and AI-assisted multimodal analysis have shifted IS research from static description to dynamic functional prediction. These tools support both mechanistic studies and translational assessment of IS-targeted therapies.

### 6.1 High-resolution and dynamic imaging

Super-resolution microscopy (SRM) overcomes the ~200 nm diffraction limit. Stimulated emission depletion microscopy (STED) and stochastic optical reconstruction microscopy (STORM) resolve F-actin reorganization and lytic granule transit in NK cell ISs (217). They also reveal PD-1 nanoclusters segregated from TCR microclusters (56). Structured illumination microscopy (SIM) improves 3D resolution for rapid whole-cell IS imaging (185). Expansion microscopy combined with lattice light-sheet microscopy (ExLLSM) enables nanoscale 3D reconstruction of intact tissues with molecular contrast (218). Early confocal work showed agrin co-localizing with CD3 at the T cell cap.

For dynamics, total internal reflection fluorescence microscopy (TIRFM) captures membrane protein diffusion and clustering within ~100 nm of the membrane. PD-1 microclusters show prolonged dwell times at inhibitory synapses, explaining the kinetic basis of PD-1-mediated negative regulation (5). LLSM provides sub-second volumetric imaging with low phototoxicity; it acquires a CTL–target conjugate in 1.3 s over hundreds of time points (219). Near-infrared-II (NIR-II) labeling permits *in vivo* tracking at depths >6 mm, enabling real-time observation of T cell–tumor contacts. These modalities allow four-dimensional (4D) tracking of IS assembly, signaling, and effector phases, and can assess synaptic quality in CAR-T and TCR-T cells.

### 6.2 *In situ* molecular interaction mapping

Förster resonance energy transfer (FRET) reports protein–protein interactions in living cells. It has confirmed myosin IIA–lytic granule association in NK cell ISs (102). Labeling Immune Partnerships by SorTagging Intercellular Contact (LIPSTIC) uses enzymatic tagging to covalently label receptor–ligand pairs during cell contact, followed by flow cytometry readout (220). Its modular design allows measurement of transient interactions (e.g., CD40–CD40L, CD28–B7) *in vitro* and *in vivo*. It is useful for screening receptor–ligand pairs that promote stable IS formation in engineered T cells.

### 6.3 Multimodal integration and artificial intelligence-assisted analysis

No single modality captures the IS regulatory network. SRM with LLSM links TCR signaling to cytoskeletal remodeling at nanoscale resolution and second-level timescales (219). FRET with SIM correlates molecular interactions with ultrastructure (102). Imaging kinetics combined with single-cell multi-omics enables closed-loop analysis from dynamic phenotypes to population validation.

AI tools include lens-free shadow imaging with deep learning for continuous cell morphology monitoring (221), K-means clustering of migration trajectories, and convolutional neural networks (CNNs) for IS image segmentation and feature extraction (220). These approaches remain discovery-stage.

### 6.4 Translational barriers to advanced IS imaging and AI

Advanced IS imaging modalities—super-resolution microscopy, live-cell tracking, and FRET- or LIPSTIC-based interaction mapping—together with AI-assisted analysis remain discovery-stage technologies (220, 222). Three barriers constrain clinical translation: standardization, validation, and reproducibility.

No consensus standard operating procedure (SOP) exists for IS induction, conjugate enrichment, fixation/permeabilization, fluorophore selection, acquisition, or live imaging. IS architecture is sensitive to antigen density, co-culture ratio, ECM stiffness, metabolic state, temperature, and time (69). Cross-platform comparisons are confounded by point spread functions, deconvolution, detector sensitivity, photobleaching, and phototoxicity. Minimum information standards, shared reference specimens, proficiency testing, and cross-laboratory calibration are needed.

Most published IS imaging and AI studies are single-center, retrospective, and underpowered. They report associations with surrogate readouts rather than pre-specified clinical endpoints (221). AI models are vulnerable to overfitting, annotation bias, class imbalance, dataset shift, and center/batch effects. External validation on independent cohorts is rare (15). Clinical deployment of an IS-derived assay would require a defined context of use; analytical validation covering repeatability, intermediate precision, reproducibility, accuracy, dynamic range, and limits of detection/quantification; and clinical validation demonstrating prognostic or predictive utility, incremental value over established biomarkers, and impact on treatment decisions. Regulatory qualification would further require traceable reference standards, audit trails, version-controlled code, and post-deployment drift monitoring. Prospective multi-center studies with pre-specified endpoints are therefore essential. Notably, machine-learning-based CAR-T IS quality quantification has shown promise as a composite biomarker correlating with clinical outcomes in multiple myeloma trials, yet has not been prospectively locked or externally validated (15).

Reproducibility is hindered by fragmented pipelines and limited data/code sharing. Segmentation, tracking, feature extraction, and clustering are parameter-dependent; different software can yield divergent results. Deep-learning models often lack interpretability. The field should adopt FAIR data principles, open code, containerized workflows, standardized metadata, blinded multi-center ring studies, and independent replication. AI models should be explainable, locked, and monitored for drift (222).

Advanced IS imaging and AI are not yet standalone clinical biomarkers. They are fit-for-purpose tools that may complement established flow cytometry, transcriptomic, and spatial biomarkers. Initial roles include pharmacodynamic monitoring in early-phase trials, stratification enrichment, and potency assessment in cell-product manufacturing. A stepwise path is realistic: standardization, then analytical and clinical validation, then regulatory qualification and integration into treatment decisions.

## 7 Challenges and future perspectives

Although therapeutic strategies targeting the IS have shown considerable promise in preclinical studies and early-phase clinical trials, their translation into clinical practice continues to face fundamental obstacles. Pronounced tumor heterogeneity hampers IS-mediated recognition, while excessive IS activation or off-target effects give rise to significant immunotoxicity. Striking a balance between restoring effective antitumor IS function and avoiding systemic autoimmune reactions remains a central challenge in this field. This section addresses these issues from three perspectives: tumor heterogeneity, immunotoxicity, and future precision regulatory strategies, with an emphasis on translation-oriented insights.

### 7.1 Tumor heterogeneity and IS recognition barriers

Intratumoral heterogeneity (ITH) constitutes a primary driver of IS-mediated immune recognition failure and tumor immune escape. At the genetic level, distinct tumor subclones carry divergent mutational profiles, resulting in inconsistent neoantigen expression. At the phenotypic level, tumor cells can actively attenuate IS initiation signals by downregulating major histocompatibility complex class I (MHC I) molecules, losing target antigens, or upregulating inhibitory ligands (117). For instance, in melanoma and non-small cell lung cancer, heterogeneous programmed death-ligand 1 (PD-L1) expression leaves certain tumor regions devoid of binding sites for checkpoint inhibitors, while epigenetic silencing of MHC class I molecules directly deprives CD8^+^ T cells of the basis for recognition. This heterogeneity is particularly devastating in adoptive cell therapy: when a CAR-T cell target antigen is lost in a subset of subclones, antigen escape and disease relapse often ensue (197).

To address this bottleneck, dual-targeting and multi-targeting strategies are being rapidly developed. Tandem CAR-T cells can simultaneously recognize two or more tumor antigens. For example, a tandem CAR targeting human epidermal growth factor receptor 2 (HER2) and interleukin-13 receptor subunit alpha 2 (IL-13Rα2) has been shown to enhance F-actin accumulation and MTOC polarization within the IS through multi-antigen recognition in glioblastoma models, thereby reducing antigen escape (172). Similarly, bispecific CD20/CD19 CAR-T cells have demonstrated the ability to cover a broader range of subclones in B cell malignancies (63). However, a key challenge of multi-targeting approaches lies in the selection of antigen combinations: without a detailed understanding of tumor ITH, low-frequency subclones may still be missed. Single-cell sequencing and spatial transcriptomics offer new tools to address this issue. By performing single-cell RNA sequencing and spatial localization analysis of tumor tissues, antigen expression heterogeneity maps can be constructed, enabling the identification of stable “core antigens” as preferred targets (128). More importantly, these technologies can simultaneously resolve the expression heterogeneity of IS-related genes in T cells themselves (e.g., CD28, LAT, ZAP-70), revealing that certain T cell subsets intrinsically possess superior synapse-forming capacity, thereby guiding the selection and expansion of adoptive cell products (10, 223).

Combination with inducers of immunogenic cell death (ICD) represents another key strategy for overcoming antigen presentation defects. Chemotherapy (e.g., anthracyclines, oxaliplatin) and radiotherapy can induce tumor cells to release damage-associated molecular patterns (DAMPs), including high mobility group box 1 (HMGB1), ATP, and calreticulin. These molecules activate receptors such as Toll-like receptor 4 (TLR4) and P2X purinoceptor 7 (P2X7) on DCs, enhancing their maturation and antigen-presenting capacity (187). Consequently, localized “cold tumors” can be converted into “hot tumors”, reactivating the cross-dressing and classical cross-presentation capabilities of DCs to provide CD8^+^ T cells with a more diverse array of pMHC signals and promote IS formation. The combination of ICD induction with ICB has demonstrated synergistic effects in multiple clinical trials, although its efficacy remains constrained by tumor heterogeneity and immunosuppressive factors within the TME.

Notably, IS formation efficiency itself may be an underappreciated predictive biomarker. Recent studies indicate that the capacity of T cells to form stable ISs with target cells varies among individuals and is closely associated with clinical responses following adoptive cell therapy. Therefore, it may become feasible to assess IS formation in peripheral blood T cells ex vivo prior to treatment and integrate machine learning algorithms to predict therapeutic efficacy, thereby achieving “IS functional stratification”. This approach moves beyond conventional paradigms that rely solely on antigen expression or tumor mutational burden (TMB), shifting the focus back to the ultimate execution step of T cell effector function.

### 7.2 IS overactivation and immunotoxicity

Enhancing IS function is a double-edged sword. Immune checkpoint inhibitors restore T cell activity by releasing inhibitory signals, but excessive release can lead to aberrant IS assembly in normal tissues, triggering immune-related adverse events (irAEs). Colitis, hepatitis, and pneumonitis associated with PD-1/PD-L1 inhibitors, as well as hypophysitis associated with CTLA-4 inhibitors, are essentially the result of aberrant IS formation in normal tissues lacking tumor antigens (52). The incidence and severity of these toxicities are directly related to the intensity of IS activation, suggesting that simply amplifying IS signaling is not the optimal strategy (54).

Off-target toxicity of CAR-T cells arises from low-level expression of target antigens in normal tissues. Even extremely low antigen density is sufficient to trigger IS formation and cytokine release under the high affinity and strong signal amplification of CARs, leading to severe adverse events. For example, long-term B cell aplasia induced by CD19 CAR-T is considered manageable, but HER2 CAR-T has caused fatal pulmonary toxicity due to low-level HER2 expression on pulmonary epithelium (137). Furthermore, cytokine release syndrome (CRS), one of the most common severe toxicities of CAR-T therapy, is fundamentally the result of systemic activation of macrophages and endothelial cells driven by amplified IS signaling (9, 185).

In response to the risk of IS overactivation, controllable CAR designs are emerging as an important direction for next-generation engineered immune cells. Drug-inducible CARs (e.g., “ON-switch” CARs based on small-molecule ligands) are split into two components that assemble into a functional CAR and initiate IS signaling only in the presence of an exogenous small molecule, thereby enabling spatial and temporal control of activation (224). Synthetic Notch (SynNotch)-based logic-gated CARs require T cells to first recognize one antigen to induce reporter gene expression, and only after recognizing a second antigen is killing triggered, significantly reducing off-target toxicity. In addition, universal CAR platforms (e.g., split, universal, and programmable chimeric antigen receptor [SUPRA CAR]) separate the antigen recognition domain from the signaling domain and use adaptor molecules to achieve multi-antigen logic gating and dose modulation, providing possibilities for precise IS initiation in complex antigen environments (173, 197).

At the clinical management level, biomarker-based patient stratification represents a practical means of mitigating immunotoxicity. PD-L1 expression levels, TMB, TME metabolic features (e.g., lactate concentration, adenosine levels), and baseline peripheral cytokine profiles have all been used to predict ICB efficacy and irAE risk (187, 189). However, the predictive power of existing biomarkers remains limited, and there is an urgent need to integrate IS functional indicators (e.g., TCR microcluster formation capacity, LFA-1 activation status) into more precise stratification models. Moreover, the development of “smart” CAR-T cells capable of sensing the microenvironment and adaptively modulating signal intensity may allow full IS activation within tumors while remaining quiescent in normal tissues, fundamentally resolving the toxicity issue.

### 7.3 Delivery barriers and the efficacy-toxicity-delivery trilemma

The central challenge of IS-targeted therapy is not merely whether a signal can be enhanced, but whether it can be delivered to the right cell, at the right time, with the right intensity, and without breaking tolerance (74). Molecular agents must overcome vascular, stromal, and TME barriers; cellular products must infiltrate, persist, and form synapses in solid tumors; multi-component systems must achieve efficient gene delivery, stable expression, and controllable activation. This creates a delivery–efficacy–toxicity trilemma: increasing affinity or signal amplification improves efficacy but worsens off-target toxicity and CRS; logic gating reduces toxicity but adds delivery complexity and failure modes; multi-targeting overcomes antigen heterogeneity but increases normal-tissue risk (171).

Delivery efficiency may already be the dominant bottleneck for several IS-targeted modalities (171). For intravenously administered nanocarriers, the median tumor accumulation is only approximately 0.7% of the injected dose, with substantial inter-study variability; thus, the vast majority of the payload distributes off-target. For CAR-T cells in solid tumors, tumor infiltration and functional IS formation are consistently low—clinical biopsies detect CAR-T cells in only a minority of patients, and preclinical models report single-digit infiltration rates (15). For bispecific antibodies, systemic dosing inevitably engages CD3 on peripheral and lymphoid T cells, reducing intratumoral activity and contributing to cytokine release syndrome; however, quantitative patient biodistribution data remain scarce (185). These constraints indicate that future IS-targeted strategies must co-optimize molecular potency with local delivery, conditional activation, PK/PD-guided dosing, and IS pharmacodynamic monitoring—although the latter remains discovery-stage and requires analytical and clinical qualification. For example, intratumoral or peritumoral delivery may improve the therapeutic index of nanocarrier-based IS modulators, while CAR-T cells may require added stromal-penetration engineering (e.g., heparanase overexpression or CXCR3 co-expression) alongside IS-enhancing modifications (153, 154).

## 8 Conclusion and perspectives

The IS is now viewed as a key functional platform that initiates, sustains, and executes antitumor immune responses. Rather than acting as a passive contact site, the IS coordinates antigen recognition, co-signal processing, cytoskeletal polarization, metabolic adaptation, and mechanical sensing within one dynamic nanomachine (225). Evidence reviewed here indicates that IS integrity is closely associated with effective antitumor immunity and may influence therapeutic outcomes; it causally influences therapeutic outcomes in immune checkpoint blockade, adoptive cell therapy, and bispecific antibody approaches (226). The IS should therefore be regarded not only as a mechanistic hub but also as a central therapeutic target in cancer immunology.

A central conclusion from this review is that IS dysfunction in tumors rarely results from a single molecular lesion. Instead, the TME disrupts IS function through multiple, overlapping mechanisms. These include metabolic competition that lowers glucose availability and increases lactate and adenosine levels (105); immunosuppressive lipids such as lysophosphatidic acid that impair calcium signaling and actin remodeling; changes in the glycocalyx and lipid rafts that alter receptor organization (227); and physical constraints caused by fibrotic stroma and altered tumor cell mechanics that weaken mechanical sensing (69).

Because so many inhibitory pathways converge on the IS, therapies aimed at only one pathway often produce incomplete or short-lived responses. Restoring IS function will therefore require combination regimens that simultaneously address metabolic suppression, adhesion and cytoskeletal defects, and aberrant biophysical properties of the TME.

At the same time, the TME still contains positive signals that could be exploited therapeutically. Antigen cross-dressing by myeloid cells, cytokine support from specific CAF subsets, intracellular complement-driven metabolic reprogramming, and chemokine gradients that stabilize the synapse all provide endogenous opportunities to rescue T cell effector function (140). The clinical goal is not only to inhibit negative signals but also to rebalance competing forces at the synaptic interface (33).

The therapeutic strategies discussed in this review—including LFA-1 allosteric agonists, cholesterol depletion, phase-separated CAR signaling condensates, and PDZbm-scaffold-engineered CAR-T cells—demonstrate that IS biology is being translated rapidly into clinical tools (228). However, current limitations are also informative. Tumor heterogeneity continues to limit therapies directed against a single antigen, and strong IS activation can cause systemic immunotoxicity (229). These two problems reveal a central challenge for IS-directed therapy: how to enhance synapse function inside tumors while avoiding inappropriate synapse formation in healthy tissues (230).

We suggest that this tension may be addressed, at least in part, by moving toward synapse programming.

The IS should be treated as an integrated regulatory node rather than a set of separate molecular pathways. Mechanical stiffness, membrane lipid composition, phase separation dynamics, and neuro-immune crosstalk are all important for IS function but remain largely untapped therapeutically (7). For instance, cholesterol depletion can change target cell membrane rigidity and improve IS formation without adding exogenous immune activators, potentially reducing the cytokine storm associated with excessive biochemical stimulation (69). Likewise, modulating sympathetic or parasympathetic signaling may provide a systemic method to adjust T cell synaptic competence without directly manipulating cells (231). Future platforms should therefore co-optimize biochemical, biophysical, and neural parameters instead of targeting only one axis.

Clinical translation of synapse programming faces three barriers (226). First, clonal antigen heterogeneity permits antigen escape after single-target programming, and trogocytosis removes CAR molecules from CAR-T cells (229). Tandem CARs partially overcome this, but their design still requires single-cell antigen maps (200). Second, IS signal strength and systemic toxicity are non-linear (230). Checkpoint inhibitors cause irAEs, CAR-T causes immune effector cell-associated neurotoxicity syndrome (ICANS), and BiTEs cause severe CRS and ICANS (185, 230). Logic-gated CARs reduce off-target effects but add failure points (232). Tumor-local delivery may overcome this. The Lymphotoxin-related inducible ligand that competes for glycoprotein D binding to herpesvirus entry mediator on T cells (LIGHT) & PαCD3 vector expresses LIGHT and membrane-anchored anti-CD3 scFv under a telomerase reverse transcriptase (TERT) promoter, remodels the ECM, builds artificial ISs, and enhances ICB and CAR-T efficacy without overt systemic toxicity (178). Third, biophysical intervention has a narrow window (69). Matrix stiffness of 10–100 kPa favors IS assembly; values outside this range impair CTL function (22). Tumor cell softening weakens LFA-1 mechanosensing and perforin pore formation (23, 70). Cholesterol depletion stiffens cancer cells and enhances adoptive T-cell efficacy in preclinical models, but its systemic effects remain untested (69). Together, these barriers indicate that no single strategy suffices. Multi-target recognition, conditional activation, local delivery, and combined biophysical-biochemical programming are needed.

IS dysfunction is not uniform across tumor types, and this variability should inform therapeutic design. In CLL, T cell dysfunction is a hallmark feature that includes defective IS formation, reduced cytotoxicity, and metabolic exhaustion (126). The mechanisms driving these defects overlap only partially with those in solid tumors, and CLL T cells exhibit distinct vulnerabilities in proximal TCR signaling and phosphatase recruitment (186). In multiple myeloma, the T cell–myeloma IS has emerged as a tractable interface for biomarker development; the frequency and transcriptional state of tumor-reactive T cells at baseline predict response to bispecific T cell engagers and quadruplet therapy, suggesting that IS competence is a host-intrinsic determinant of therapeutic efficacy. In pancreatic cancer, CAF-mediated inhibitory synapses with Tregs create a locally immunosuppressive network that is less prominent in melanoma or NSCLC, where vascular ICAM-1 polarization and tumor-cell-intrinsic ICAM-1 downregulation dominate instead (132). These tumor-type-specific differences have two consequences. First, the same IS-targeted agent may show efficacy in one tumor type but not another, depending on which disruption axis dominates. IrAE risk varies by tumor type and immunotherapy class; hepatitis is frequently associated with immune-cytokine treatment, while T cell bispecifics produce organ-specific toxicity profiles (230). From a personalized medicine perspective, IS functional stratification should therefore be tumor-type-informed. Pre-treatment assessment of synaptic competence—TCR microcluster assembly, LFA-1 activation dynamics, or lytic granule polarization efficiency—could complement established biomarkers such as TMB and PD-L1 (15). Establishing tumor-type-specific reference ranges for IS functional parameters will require multi-institutional consortia with standardized protocols. Combining imaging-based IS readouts with single-cell transcriptomic and spatial profiling of the TME could then support IS-informed precision immunotherapy, in which patients receive treatments matched to their specific synaptic defects. Machine-learning tools for CAR IS quality quantification, already shown to correlate with clinical outcomes in multiple myeloma trials, provide a template for this approach, though prospective locking and external validation remain necessary (15).

Future engineered immune cells should contain built-in sensing and gating systems that enable context-dependent IS activation. Logic-gated CARs, small-molecule-controlled receptors, and universal adaptor platforms are early examples (232). More advanced designs are expected to include feedback loops that detect local metabolic or mechanical signals, allowing complete synaptic activation in tumors while keeping cells quiescent in normal tissues. Such “smart synapse” engineering could separate efficacy from toxicity and help resolve a major clinical dilemma (233). Advanced imaging and AI provide the verification layer for synapse programming. Multimodal platforms integrating super-resolution imaging, lattice light-sheet tracking, FRET-based interaction mapping, and deep learning can track IS dynamics at nanoscale resolution and second-level timescales (219). These tools will be critical for developing predictive models of IS function that inform both the design and clinical use of synapse-directed therapies (222).

In summary, the IS sits at the intersection of tumor biology, immunotherapy, and emerging technologies. Progress will require moving beyond a molecular parts list of IS components and toward a systems-level view of synaptic function that includes metabolism, mechanics, phase separation, and neural regulation. The future of IS-directed cancer therapy will depend on both repairing defective synapses and re-engineering them with precision, selectivity, and adaptability.

## Glossary

4-1BB: tumor necrosis factor receptor superfamily member 9 (CD137)
4-1BBL: 4-1BB ligand (CD137L)
A2A: adenosine A2A receptor
A2B: adenosine A2B receptor
ACAT1: acetyl-CoA acetyltransferase 1
AhR: aryl hydrocarbon receptor
AKT: protein kinase B
ALCAM: activated leukocyte cell adhesion molecule
APC: antigen-presenting cell
Arp2/3: actin-related protein 2/3 complex
ATG7: autophagy-related protein 7
ATP: adenosine triphosphate
B7-H3: B7 homolog 3
BCR: B cell receptor
BiTE: bispecific T-cell engager
BTN2A1: butyrophilin subfamily 2 member A1
BTN3A1: butyrophilin subfamily 3 member A1
Btk: Bruton’s tyrosine kinase
C3: complement component 3
C3a: complement component 3a
C3aR: complement component 3a receptor
C3b: complement component 3b
C3d: complement component 3d
CAF: cancer-associated fibroblast
CAR: chimeric antigen receptor
CAR-NK: chimeric antigen receptor natural killer cell
CAR-T: chimeric antigen receptor T cell
CCL22: C-C motif chemokine ligand 22
CCR7: C-C chemokine receptor type 7
CD2: cluster of differentiation 2
CD3: cluster of differentiation 3
CD3ϵ: cluster of differentiation 3 epsilon chain
CD16: cluster of differentiation 16
CD19: cluster of differentiation 19
CD20: cluster of differentiation 20
CD27: cluster of differentiation 27
CD28: cluster of differentiation 28
CD36: cluster of differentiation 36
CD39: cluster of differentiation 39
CD40: cluster of differentiation 40
CD40L: cluster of differentiation 40 ligand
CD45: cluster of differentiation 45
CD46: cluster of differentiation 46
CD58: cluster of differentiation 58
CD70: cluster of differentiation 70
CD73: cluster of differentiation 73
CD80: cluster of differentiation 80
CD86: cluster of differentiation 86
CD99: cluster of differentiation 99
CD137: cluster of differentiation 137
CD166: cluster of differentiation 166
CD200: cluster of differentiation 200
CD226: cluster of differentiation 226
CD276: cluster of differentiation 276
CD277: cluster of differentiation 277
cDC1: conventional dendritic cell type 1
cDC2: conventional dendritic cell type 2
CGRP: calcitonin gene-related peptide
CHRM2: cholinergic receptor muscarinic 2
CLDN18: the tight-junction protein claudin 18
CLL: chronic lymphocytic leukemia
CNN: convolutional neural network
CR2: complement receptor type 2
CR3: complement receptor type 3
CRS: cytokine release syndrome
cSMAC: central supramolecular activation cluster
CTL: cytotoxic T lymphocyte
CTLA-4: cytotoxic T-lymphocyte-associated protein 4
CXCL9: C-X-C motif chemokine ligand 9
CXCL10: C-X-C motif chemokine ligand 10
CXCR3: C-X-C chemokine receptor type 3
Cx43: connexin 43
DAG: diacylglycerol
DAMPs: damage-associated molecular patterns
DC: dendritic cell
DGKζ: diacylglycerol kinase zeta
DLL3: delta-like ligand 3
DNMT: DNA methyltransferase
DNMT1: DNA methyltransferase 1
DOCK8: dedicator of cytokinesis 8
dSMAC: distal supramolecular activation cluster
EPAC1: exchange protein directly activated by cAMP 1
ER: endoplasmic reticulum
ExLLSM: expansion microscopy combined with lattice light-sheet microscopy
F-actin: filamentous actin
FcR: Fc receptor
FcγRIIB: Fc gamma receptor IIB
FcRH5: Fc receptor-like protein 5
FIP3: Rab11 family-interacting protein 3
Flt3L: FMS-like tyrosine kinase 3 ligand
FMRP: fragile X messenger ribonucleoprotein protein
FoxO: forkhead box O
FRET: Förster resonance energy transfer
GM-CSF: granulocyte-macrophage colony-stimulating factor
GSDME: gasdermin E
HIF-1α: hypoxia-inducible factor 1 alpha
HMGB1: high mobility group box 1
HSV-1: herpes simplex virus type 1
iCAF: inflammatory CAF
ICAM-1: intercellular adhesion molecule 1
ICANS: immune effector cell-associated neurotoxicity syndrome
ICB: immune checkpoint blockade
ICD: immunogenic cell death
IDO: indoleamine 2,3-dioxygenase
IFN-γ: interferon gamma
IFITM3: interferon-induced transmembrane protein 3
IL-1β: interleukin 1 beta
IL-2: interleukin 2
IL-6: interleukin 6
IL-10: interleukin 10
IL-12: interleukin 12
IL-15: interleukin 15
IL-21: interleukin 21
IL-27: interleukin 27
IP3R1: inositol 1,4,5-trisphosphate receptor type 1
IPP: isopentenyl pyrophosphate
irAE: immune-related adverse event
IRF7: interferon regulatory factor 7
IS: immune synapse
ITAM: immunoreceptor tyrosine-based activation motif
ITIM: immunoreceptor tyrosine-based inhibitory motif
ITH: intratumoral heterogeneity
ITT: immunoglobulin tail tyrosine
iC3b: inactivated complement component 3b
JNK: c-Jun N-terminal kinase
Kyn: kynurenine
LAG-3: lymphocyte activation gene 3
LAMP-1: lysosome-associated membrane protein 1
LAMP2: lysosome-associated membrane protein 2
LAT: linker for activation of T cells
LFA-1: lymphocyte function-associated antigen 1
LIGHT: Lymphotoxin-related inducible ligand that competes for glycoprotein D binding to herpesvirus entry mediator on T cells
LIPSTIC: labeling immune partnerships by SorTagging intercellular contact
LLT1: lectin-like transcript 1
LLSM: lattice light-sheet microscopy
LPA: lysophosphatidic acid
LYST: lysosomal trafficking regulator
MAPT: microtubule-associated microtubule-associated protein tau
MCT1: monocarboxylate transporter 1
MCT4: monocarboxylate transporter 4
mDia1: mammalian diaphanous 1
MGAT5: mannosylglycoprotein β-1,6-N-acetylglucosaminyltransferase 5
MHC: major histocompatibility complex
MHC I: major histocompatibility complex class I
MHC II: major histocompatibility complex class II
miRNA: microRNA
MTOC: microtubule-organizing center
mTOR: mechanistic target of rapamycin
mTORC1: mechanistic target of rapamycin complex 1
MYH9: myosin heavy chain 9
NAA: N-acetylaspartate
NAT8L: N-acetyltransferase 8-like
NIR-II: near-infrared-II
NK: natural killer cell
NKG2D: natural killer group 2 member D
NKp30: natural cytotoxicity triggering receptor 3
NKp46: natural cytotoxicity triggering receptor 1
NKR-P1: natural killer receptor protein 1
NLRP3: NLR family pyrin domain containing 3
Nrp1: neuropilin-1
OX40L: OX40 ligand
OXPHOS: oxidative phosphorylation
P2X7: P2X purinoceptor 7
p66Shc: 66 kDa Src homology 2 domain-containing protein
PC: pyruvate carboxylase
PD-1: programmed cell death protein 1
PD-L1: programmed death-ligand 1
PDH: pyruvate dehydrogenase
PDZ: postsynaptic density protein-95/discs large/zonula occludens-1
PDZbm: PDZ-binding motif
PGE2: prostaglandin E2
PI3K: phosphoinositide 3-kinase
PI3Kγ: phosphoinositide 3-kinase gamma
PIP3: phosphatidylinositol-3,4,5-trisphosphate
PKA: protein kinase A
PKC-θ: protein kinase C theta
pMHC: peptide-major histocompatibility complex
pSMAC: peripheral supramolecular activation cluster
PTEN: phosphatase and tensin homolog
Rab11: Ras-related protein Rab-11
Rab27a: Ras-related protein Rab-27A
Rac: Ras-related C3 botulinum toxin substrate
RasGRP1: RAS guanyl releasing protein 1
RhoA: Ras homolog family member A
RhoB: Ras homolog family member B
sCD58: soluble CD58
Sema4A: semaphorin 4A
SHP-1: Src homology region 2 domain-containing phosphatase-1
sHDL: synthetic high-density lipoprotein
Siglec-7: sialic acid-binding immunoglobulin-like lectin 7
Siglec-9: sialic acid-binding immunoglobulin-like lectin 9
SIM: structured illumination microscopy
SLAMF6: signaling lymphocytic activation molecule family member 6
SLP76: SH2 domain-containing leukocyte protein of 76 kDa
SMAP: supramolecular attack particle
SNARE: soluble N-ethylmaleimide-sensitive factor attachment protein receptor
SNX27: sorting nexin 27
SP: substance P
STAT1: signal transducer and activator of transcription 1
STAT3: signal transducer and activator of transcription 3
STAT4: signal transducer and activator of transcription 4
STAT5: signal transducer and activator of transcription 5
STED: stimulated emission depletion microscopy
STORM: stochastic optical reconstruction microscopy
SUCNR1: succinate receptor 1
SUN2: Sad1/UNC-84 homology 2
SUPRA CAR: split, universal, and programmable chimeric antigen receptor
SynNotch: synthetic Notch
TAA: tumor-associated antigen
TAM: tumor-associated macrophage
T-bet: T-box transcription factor TBX21
TCE: T cell engager
TCR: T cell receptor
TCR-T: T cell receptor engineered T cell
TERT: telomerase reverse transcriptase
TGF-β: transforming growth factor beta
Th1: T helper type 1
Th2: T helper type 2
Th17: T helper type 17
TIGIT: T cell immunoreceptor with immunoglobulin and ITIM domains
TIM-3: T cell immunoglobulin and mucin domain-containing protein 3
TIRFM: total internal reflection fluorescence microscopy
TLR: Toll-like receptor
TLR4: Toll-like receptor 4
TLR9: Toll-like receptor 9
TMB: tumor mutational burden
TME: tumor microenvironment
TNF-α: tumor necrosis factorα
Treg: regulatory T cell
TRUCK: T cells redirected for universal cytokine killing
TRX1: thioredoxin-1
TSA: tumor-specific antigen
TSP-1: thrombospondin-1
TSP-4: thrombospondin-4
Tpres: T presenting cell
UHRF1: ubiquitin-like with PHD and ring finger domains 1
Vamp-7: vesicle-associated membrane protein 7
Vav1: vav guanine nucleotide exchange factor 1
Vγ9Vδ2: V gamma 9 V delta 2 T cell
WASp: Wiskott-Aldrich syndrome protein
WAVE: WASP-family verprolin homologous protein
WRC: WAVE regulatory complex
ZAP-70: zeta-chain-associated protein kinase 70.
